# Allele-Specific Chromatin Immunoprecipitation Studies Show Genetic Influence on Chromatin State in Human Genome

**DOI:** 10.1371/journal.pgen.0030081

**Published:** 2007-05-18

**Authors:** Mitsutaka Kadota, Howard H Yang, Nan Hu, Chaoyu Wang, Ying Hu, Philip R Taylor, Kenneth H Buetow, Maxwell P Lee

**Affiliations:** 1 Laboratory of Population Genetics, Center for Cancer Research, National Cancer Institute, National Institutes of Health, Bethesda, Maryland, United States of America; 2 Genetic Epidemiology Branch, Division of Cancer Epidemiology and Genetics, National Cancer Institute, National Institutes of Health, Bethesda, Maryland, United States of America; Massachusetts General Hospital, United States of America

## Abstract

Several recent studies have shown a genetic influence on gene expression variation, including variation between the two chromosomes within an individual and variation between individuals at the population level. We hypothesized that genetic inheritance may also affect variation in chromatin states. To test this hypothesis, we analyzed chromatin states in 12 lymphoblastoid cells derived from two Centre d'Etude du Polymorphisme Humain families using an allele-specific chromatin immunoprecipitation (ChIP-on-chip) assay with Affymetrix 10K SNP chip. We performed the allele-specific ChIP-on-chip assays for the 12 lymphoblastoid cells using antibodies targeting at RNA polymerase II and five post-translation modified forms of the histone H3 protein. The use of multiple cell lines from the Centre d'Etude du Polymorphisme Humain families allowed us to evaluate variation of chromatin states across pedigrees. These studies demonstrated that chromatin state clustered by family. Our results support the idea that genetic inheritance can determine the epigenetic state of the chromatin as shown previously in model organisms. To our knowledge, this is the first demonstration in humans that genetics may be an important factor that influences global chromatin state mediated by histone modification, the hallmark of the epigenetic phenomena.

## Author Summary

Human health and disease are determined by an interaction between genetic background and environmental exposures. Both normal development and disease are mediated by epigenetic regulation of gene expression. The epigenetic regulation causes heritable changes in gene expression, which is not associated with DNA sequence changes. Instead, it is mediated by chemical modification of DNA such as DNA methylation or by protein modifications such as histone acetylation and methylation. Although much has been known about epigenetic inheritance during development, little is known about the influence of the genetic background on epigenetic processes such as histone modifications. In this report the authors studied five histone modifications on a genome-wide level in cells from different families. Global epigenetic states, as measured by these histone modifications, showed a similar pattern for cells derived from the same family. This study demonstrates that genetic inheritance may be an important factor influencing global chromatin states mediated by histone modifications in humans. These observations illustrate the importance of integrating genetic and epigenetic information into studies of human health and complex diseases.

## Introduction

Polymorphisms and quantitative differences in gene expression provide the genetic basis for human variation. Studies in humans and other organisms suggest that variation at the transcript level accounts for the majority of the phenotypic variation among species and across individuals within species [1–4]. Recent studies have demonstrated that inherited factors influence gene expression variation between both copies of a gene within an individual [5] as well as between individuals [1,6–8]. In a large-scale analysis of allele-specific gene expression using Affymetrix HuSNP chip [9], we found that allelic variation in gene expression is common, affecting about half of the genes in human genome. This conclusion was supported from the studies of digital gene expression in UniGene database [10,11] and allele-specific gene expression using a custom-designed single nucleotide polymorphism (SNP) chip [12]. Analysis of allelic variation in gene expression can facilitate identification of regulatory SNPs when the regulatory SNPs are in linkage disequilibrium with an exonic SNP used in the analyses of allele-specific gene expression [13,14].

Eukaryotic genomes are organized into chromatin, formed by DNA and protein complex. The basic unit of chromatin is the nucleosome structure containing 146 bp DNA that wraps around a histone octamer. At the chromosome level, gene expression is regulated by distinct chromatin structures. This epigenetic information is often encoded in post-translational modifications of histone proteins such as acetylation, methylation, and phosphorylation [15]. Histone modifications can be maintained through mitotic cell divisions. This stable transmission of epigenetic state through mitosis provides the basis for cellular differentiation and organism development. Although there are a few examples of inheritance of epigenetic information across generations in model organisms [16,17], no investigation of the global effect of genetic inheritance on chromatin state in humans has been reported. In light of genetic influence on allelic gene expression variation in pedigree [5], we set out to analyze if genetic inheritance also affects chromatin variation in humans, as measured by variations in histone modifications using an allele-specific chromatin immunoprecipitation (haploChIP) assay [13]. The use of allele-specific variation in chromatin state in a heterozygous individual is a powerful approach to study genetic influence since other sources of variations in the cellular environment are likely affecting both alleles more or less equally. Our study demonstrates that specific chromatin states as a quantitative trait show familial aggregation.

## Results

### Allele-Specificity of Chromatin Immunoprecipitation Assay

**Figure 1:**
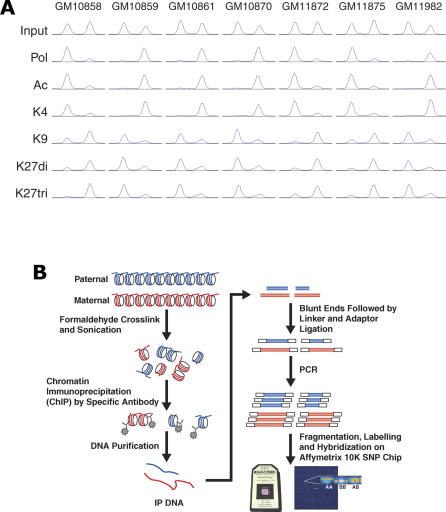
Allele-Specific ChIP Assay and Modification of 10K SNP ChIP Protocol for ChIP-on-chip Experiments (A) The haploChIP assay shows allele-specific chromatin binding by Pol II and histone H3 protein at the differentially methylated region in the imprinted *LIT1* promoter. We used allele-specific OLA to detect allele-specific ChIP activities. The two peaks represent allele-specific ChIP activities at the C allele (left) and the T allele (right). GM10858, GM10859, GM10861, GM10870, GM11872, GM11875, and GM11982 are seven CEPH samples from CEPH pedigrees family identification 1347 and 1362 that are heterozygous at SNP (rs11023840). input, DNA from whole cell extract; Pol, RNA polymerase II; Ac, H3Ac; K4, H3K4; K9, H3K9; K27di, H3K27 dimethylation; K27tri, H3K27 trimethylation. (B) Our modified 10K SNP chip protocol for the haploChIP-on-chip experiments is illustrated here.

To evaluate the allele-specificity of our chromatin immunoprecipitation (ChIP) assay, we first examined protein binding at three imprinted genes *(LIT1*, *H19*, and *SNRPN)* and two X-linked genes *(HPRT1* and *PGK1)* loci. We used 12 lymphoblastoid cells derived from 12 individuals from two Centre d'Etude du Polymorphisme Humain (CEPH) families. Each cell line was characterized by six antibodies targeting at chromatin proteins. The description of cell lines and experiments can be found in Tables S3, S6, S7, and S8. Three antibodies target at active chromatin proteins, which are RNA polymerase II (Pol II), histone H3 lysine 9/14 acetylation (H3Ac), and lysine 4 dimethylation (H3K4). The remaining three antibodies target at inactive chromatin proteins, which are histone H3 lysine 9 dimethylation (H3K9), lysine 27 dimethylation (H3K27di) and trimethylation (H3K27tri). The control DNAs were from whole cell extract, which were prepared as the ChIP experiments, except for the omission of antibodies. We refer to this control DNA as input. We analyzed DNAs that were co-immunoprecipitated by the antibodies using oligo ligation assay (OLA). The results for the differential methylation region in *LIT1* promoter are shown in Figure 1A. The paternal allele was specifically pulled down by antibodies targeting at active chromatin (Pol, Ac, and K4 in Figure 1A; the paternal allele is C for GM10858, GM11872, and GM11875 and T for GM10859, GM10861, GM10870, and GM11982). This is consistent with the previous study, which demonstrated that the *LIT1* gene was imprinted and was expressed from paternal chromosome only [18]. The maternal allele was preferentially pulled down by antibodies targeting at inactive chromatin (K9, K27di, K27tri in Figure 1A; the maternal allele is T for GM10858, GM11872, and GM11875 and C for GM10859, GM10861, GM10870, and GM11982). As a control, the input showed nearly equal intensities of both alleles. Promoter regions of *H19, SNRPN, HPRT1,* and *PGK1* also displayed expected allele-specificity in our ChIP assays (Figure S6).

### Clustering of Samples by Family with Chromatin State

After we have established allele-specificity for our ChIP assay using the imprinted genes and X-linked genes, we proceeded to analyze genome-wide allele-specific chromatin states by ChIP-on-chip method with a SNP chip. Since Affymetrix 10K SNP chip was designed for genotyping purpose, we had to modify the protocol in order to use the 10K SNP chip for doing ChIP-on-chip studies. The modified protocol is illustrated schematically in Figure 1B. We first repaired DNA fragments that were co-immunoprecipitated by antibodies or from the nonenriched control DNAs (input) by flushing the ends with a nuclease and adding adaptors to the DNA ends (Figure 1B). The DNA fragments were amplified and hybridized separately to the 10K SNP chips. We used 12 lymphoblastoid cell lines derived from 12 individuals, six of them from each of the two CEPH families (1347 and 1362, two parents and four children). Each cell line was analyzed with the six antibodies (Pol II, H3Ac, H3K4, H3K9, H3K27di, and H3K27tri) and two controls (input and genomic DNA using unmodified protocol), which gave 96 ChIP-on-chip experiments. The data from the 96 ChIP-on-chip experiments can be represented in a data matrix, with 96 rows (experiments) and 10,000 columns (SNPs). Each SNP had two measurements, one for chromatin binding from the A allele and the other from the B allele. We were interested in two derived values. The first one was the total intensity, which was the sum of chromatin-binding intensities from A allele plus B allele. The total intensity was similar to those obtained in conventional ChIP-on-chip experiments. The second one was the relative intensity, which was the ratio of A allele chromatin-binding intensity divided by the total intensity. The relative intensity was uniquely produced in this study due to the use of the SNP chip in ChIP-on-chip experiment. The input serves as an important control for two purposes. First, both input and the ChIP-on-chip experiment used our modified protocol. Comparison of genotype call between genomic DNA and input allowed us to evaluate the allelic specificity of our protocol for this experimental system. We found that the concordance of genotype call between genomic DNA and input was usually around 99% (Table S1). Thus, the result validated our protocol. Second, it allows us to define biological activity specifically due to chromatin beyond a baseline. The baseline can be assessed by input. Because the complexity in this high dimensional ChIP-on-chip data, we need to reduce the complexity in order to effectively understand the variance structure. We used principal component analysis (PCA) for this purpose. In our data, the first two principal components typically account for between 10%–50% of the total variance. Therefore, we can now focus our analyses in two dimensions instead of the original 10,000 dimensions.

**Figure 2:**
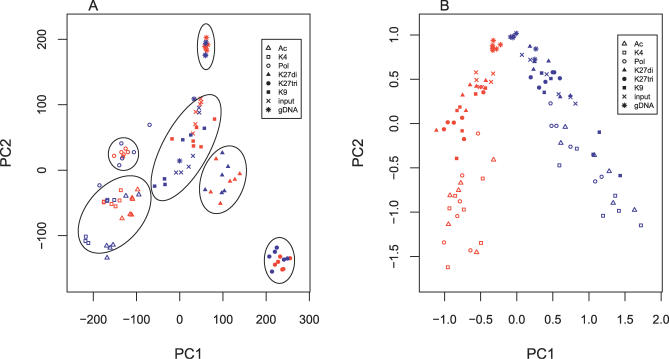
Clustering of the Samples by Antibody with Total Chromatin-Binding Activity (A + B) versus Clustering of the Samples by Family with the Relative Allelic Chromatin-Binding Activity (A/A + B) (A) PCA was performed with the total ChIP intensity (A + B) for the samples in CEPH family 1362 (red) and 1347 (blue). Ac, histone H3 lysine 9/14 acetylation; K4, histone H3 lysine 4 dimethylation; Pol, RNA polymerase II; K27di, histone H3 lysine 27 dimethylation; K27tri, histone H3 lysine 27 trimethylation; K9, histone H3 lysine 9 dimethylation; in, DNA from whole cell extract; and gDNA, genomic DNA. The clusters formed by experiments with different types of the antibodies are enclosed with ellipses. Similar PCA projection with more complete sample labeling to assist interpretation is presented in Figure S1. (B) PCA was performed with the relative binding intensity (A/A + B) using the same ChIP data as (A). Similar PCA projection with more complete sample labeling is shown in Figure S2. Color coding: CEPH 1347, red; CEPH 1362, blue. The samples are described in Table S1.

**Figure 3:**
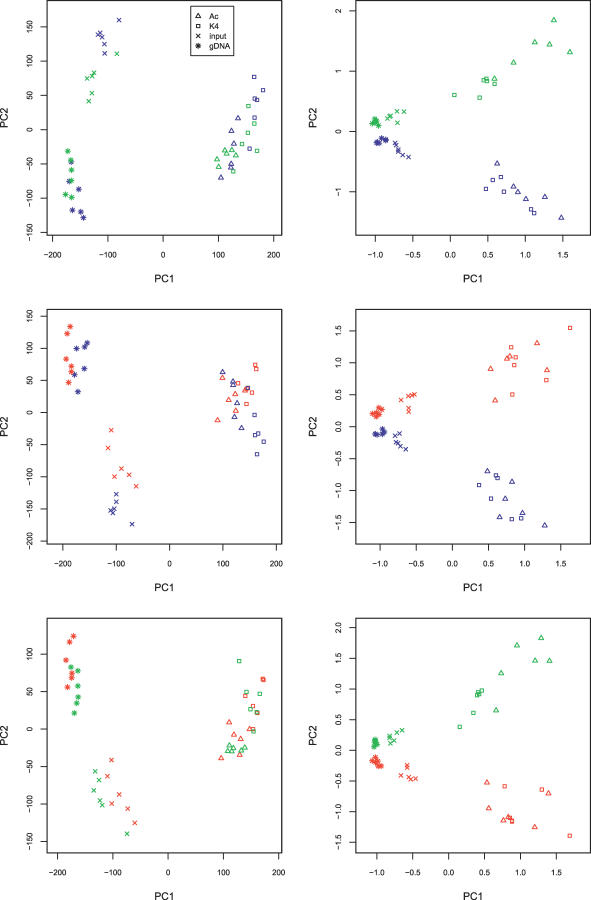
Clustering of the Samples by Family with Allelic Chromatin-Binding Activity We analyzed three pair-wise combinations of CEPH families, 1347 (red), 1331 (blue), and 1413 (green) using PCA. All data were generated using the version 2 of the 10K SNP chip. PCAs were performed with the total intensity (A + B) (figures on the left side) as well as with the relative binding intensity (A/A + B) (figures on the right side). Similar PCA projections with more complete sample labeling are shown in Figure S3, Figure S4, and Figure S5. The samples are described in Table S1. Ac, histone H3 lysine 9/14 acetylation; K4, histone H3 lysine 4 dimethylation; input, DNA from whole cell extract; gDNA, genomic DNA.

The result from PCA analysis using total intensity (A + B) for the ChIP-on-chip data is shown in Figure 2A. We plotted the 96 samples using the scores from the first principal component (PC1) and the second principal component (PC2). As shown in Figure 2A, the samples were clustered by the antibodies using the total chromatin-binding intensities (A + B). For example, the samples from antibodies targeting at active chromatin (Pol II, H3Ac, and H3K4) are on the left. Samples from two (H3K27di and H3K27tri) of the three antibodies targeting at inactive chromatin are on the right. Samples from the third antibody targeting inactive chromatin (H3K9) and the controls are in the middle. This is expected because chromatin states are determined by histone modifications and Pol II activity. Samples from the two families (red and blue, Figure 2A), are all intermixed. Therefore, we concluded that the major determinant of the total variance in the ChIP-on-chip experiments was due to variations in chromatin states as revealed by the antibodies targeting at different modification forms of histone H3 proteins and Pol II when using the total intensity. However, we got a totally different picture when PCA was performed with the relative intensity (A/A + B) (Figure 2B). Now the samples from the family 1 and family 2 (red and blue, Figure 2B) were separated from each other into two clusters. The separation was the largest for the antibodies targeting at active chromatin, which were represented by the open symbols at the bottom of Figure 2B. So the global chromatin states as measured by the relative intensity from the A allele differ for the individuals in family 1 versus the individuals in family 2. This observation led us to conclude that genetic inheritance can influence chromatin modifications. To validate this important finding, we carried out the same ChIP-on-chip experiments and analyses for two additional families (1331 and 1413). We analyzed twelve lymphoblastoid cell lines, six from each of the two CEPH families (1347 and 1362), with the two antibodies (H3Ac and H3K4) and the two controls (input and genomic DNA). Once again, we saw clustering of the samples by controls/antibody (H3Ac and H3K4) when PCA was performed using the total intensity (A + B) (Figure 3, left panels, three pair-wise comparisons among CEPH families 1347, 1331, and 1413). More importantly, samples from the two different families were separated into two clusters when PCA was analyzed using the relative intensity (A/A + B) (Figure 3, right panels, three pair-wise comparisons among CEPH families 1347, 1331, and 1413).

### Chromatin State Is Similar in Genetically Related Individuals

**Figure 4:**
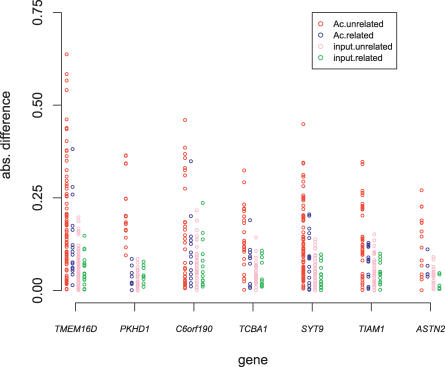
Chromatin Variation in Genetically Unrelated Pairs Is Larger Than Genetically Related Pairs Only the heterozygous individuals from the four CEPH families are analyzed here. Genetically related pairs consist of siblings or child-parent relationship. All others are genetically unrelated pairs. The SNPs analyzed here met the following three criteria: (1) the variance in the H3Ac samples was significantly large (Chi-square test, *p* < 0.05); (2) the variance in the input was small; and (3) the variance in genetically related pairs is significantly larger than the variance in genetically unrelated pairs (F-test, *p* < 0.05). The selected genes (SNPs) are *TMEM16D* (SNP ID: rs938335), *PKHD1* (SNP ID: rs1414503), *C6orf190* (SNP ID: rs270015), *TCBA1* (SNP ID: rs590944), *SYT9* (SNP ID: rs2346824), *TIAM1* (SNP ID: rs2409411), and *ASTN2* (SNP ID: rs719535). Each circle represents a pair. The absolute difference in the relative binding intensity (A/A + B) between the two individuals within the pair is plotted.

To better understand the genetic influences on chromatin variation, we constructed pairs of genetically related individuals (siblings or parent-child), as well as pairs of genetically unrelated individuals from heterozygous individuals in the four CEPH families. We then computed the difference between the two individuals in each pair. To identify those SNPs that had similar chromatin state within genetically related individuals, we compared the variance in genetically related pairs versus the variance in genetically unrelated pairs. We identified seven SNPs (F-test, *p* < 0.05). Variation in chromatin state for the seven SNPs was smaller in the genetically related pairs than the variation in the genetically unrelated pairs (Figure 4), indicating similar chromatin state between the related individuals. These differences were specifically observed in the ChIP experiment (in H3Ac but absent in input).

### Mendelian Inheritance Analysis of Chromatin State

**Figure 5:**
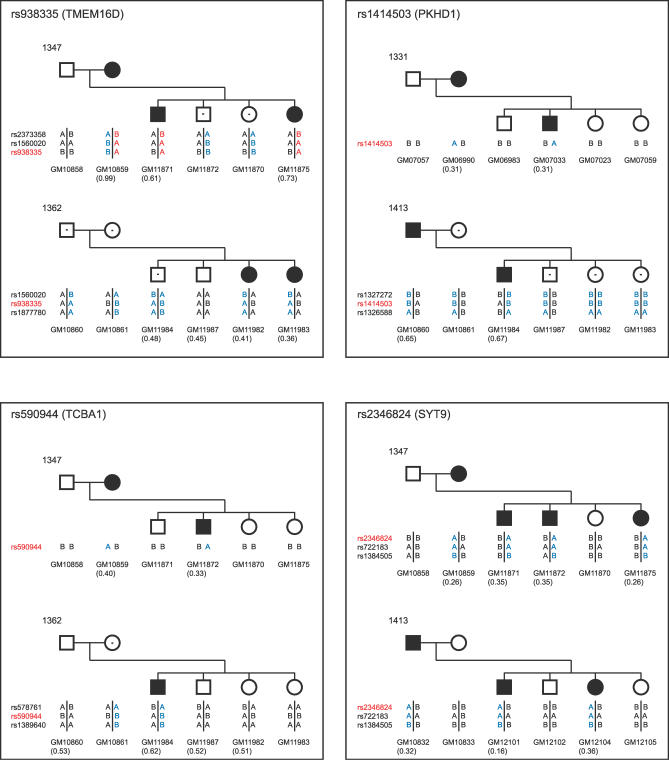
Mendelian Inheritance Analysis of Allelic-Specific Histone H3 Acetylation State Pedigree analysis was carried out for the seven SNPs analyzed in Figure 4. The results for the four genes, rs938335 *(TMEM16D),* rs1414503 *(PKHD1),* rs590944 *(TCBA1),* and rs2346824 *(SYT9)* are shown here in Figure 5, while the rest of the three genes, rs2409411 *(TIAM1),* rs270015 *(c6orf190),* and rs719535 *(ASTN2)* are shown in Figure S8. Each individual is shown with CEPH family identification, sample identification, and genetic information (SNP genotype or haplotype). The haplotype with higher chromatin-binding activity (two standard deviations above the mean) is highlighted in red, whereas the haplotype with lower chromatin-binding activity (two standard deviations below the mean) is highlighted in blue. Allelic fraction values (A/(A + B)) are also shown for heterozygous samples (>0.5, A > B; 0.5, A = B; <0.5, A < B). Filled circles or squares indicate the affected individual (allelic fraction is significantly different from 0.5), and a dot in a circle or square indicates a carrier containing a high or low allele. Genotype calls were derived from genomic DNA call from 10K SNP experiment, and A or B alleles are assigned in alphabetical order of the nucleotides (A, C, G, T) for each SNP as defined by Affymetrix calling algorithm.

Yan et al. previously demonstrated that allelic gene expression variation segregated as a Mendelian trait [5]. To evaluate if allelic chromatin variation also follows Mendelian inheritance, we performed inheritance analysis for the seven genes analyzed in Figure 4. All seven genes showed segregation patterns that were consistent with Mendelian inheritance (Figure 5 and Figure S8). For examples, ABB haplotype in GM10859 (mother in CEPH family 1347) in the case of rs938335 had low H3Ac binding activity (below two standard deviations from the mean intensity of B allele), whereas BAA haplotype in GM10859 had high H3Ac binding activity (above two standard deviations from the mean of A allele). The two heterozygous children are GM11871 and GM11875, both of whom received BAA from the mother. The allelic fraction values (A/A + B) are 0.61 and 0.73, respectively, which are higher than 0.5. But the allelic values are not as extreme as the one in GM10859. This is because the paternal allele AAB has normal level of H3Ac binding activity. Similarly, BAA and ABB haplotypes in CEPH family 1362 have low H3Ac binding activity. Therefore, GM11982 and GM11983 had low allelic fraction values, 0.41 and 0.36, respectively. However, GM11984 received both alleles that had low H3Ac binding activities. Consequently, the allelic fraction was 0.48, very close to 0.5. Conversely, GM11987 received both alleles that had normal H3Ac binding activities, thus the allelic fraction value was also close to 0.5. Note that this is different from conventional Mendelian inheritance analysis in that it uses the allelic fraction as a phenotypic trait, and this depends on relative quantities between the two alleles. Nevertheless, our results agree very well with inheritance of the chromatin state, in turn providing direct support for genetic influence on the chromatin state. However, we must qualify our results by noting that, in contrast to the relatively large number of informative individuals studied in Yan et al. [5] (eight and ten informative individuals per family for two different genes) (Figure 1), the maximum number of individuals informative for any SNP tested in any family in our study is five. This limits the statistical power of our inheritance analysis, despite a highly suggestive result.

Taken together, these results suggest that inherited genetic components could determine the epigenetic state of the chromatin. To our knowledge, this is the first demonstration in humans that genetic inheritance may be an important factor directing the global chromatin state mediated by histone modification, the hallmark of the epigenetic phenomena.

## Discussion

Our aim was to determine if genetic inheritance can influence chromatin state globally in humans. Our studies support the notion that inherited genetic components can determine the epigenetic state of the chromatin.

Our strategy was to use samples from different pedigrees to assess the genetic effect. The use of the SNP chip to measure allele-specific chromatin-binding intensity in a heterozygous individual and the use of relative binding intensity between the two alleles enables us to detect difference in chromatin state in individuals between different families since other sources of variations in the cellular environment are likely affecting both alleles more or less equally. The use of PCA made it possible to focus analyses on a few components, which have the capacity to combine weak signals from multiple genetic loci. Otherwise, the weak signal may not be detectable when analyzed individually.

We used a combination of 12 lymphoblastoid cell lines and six antibodies plus two controls in the experiment. This is two-factor experiment design. Genetic factor has two levels, one for each family; whereas chromatin factor has three levels, one for active chromatin, one for inactive chromatin, and one for control. This study design allows us to assess genetic inheritance effect as well as chromatin states targeted by the six antibodies on the total variance across the 96 experiment data. We are interested in the variance across the 96 samples. In our variance component model, we decomposed the total variance into three components, genetics, chromatin, and residual variance. Because the complexity in this high dimensional ChIP-on-chip data, we need to reduce the complexity to effectively understand the variance structure. We used PCA for this purpose. What PCA does is to transform the data matrix by rotating the coordinate system. After transformation, we have a new set of variables, denoted by principal components. Each principal component is a linear combination of the original variables. PCA has two useful mathematic properties. First, all principal components are orthogonal to each other, so the total variance is simply the sum of variances from each principal component. Second, principal components are ranked so that PC1 accounts for the largest variance in the data, followed by PC2. In our studies, the first two principal components usually account for about 10%–50% of the total variance. Therefore, we were able to focus the analyses in two dimensions instead of the original 10,000 dimensions.

PCA using total ChIP signal as (A + B) (Figures 2A and 3, panels on the left) indicated that the total variance in the samples was comprised mostly by antibodies targeting at various chromatin proteins, which also demonstrated the specificity of the ChIP assay. In contrast, PCA using the relative signal (A/A + B) indicated that the total variance in the samples comprised primarily the difference between two families and secondarily antibodies targeting at various chromatin proteins (Figures 2B and 3, panels on the right). The separation between different families in controls served as the baseline, which captured the background level of difference due to genotypes. The separation between different families is much too large for the antibodies targeting at active chromatin, indicating specific chromatin state differences between different families. This result suggests that genetic inheritance can influence the global chromatin state. The relative intensity measurement (A/A + B) has better sensitivity in detecting the genetic effect than the total intensity (A + B), since other sources of variations in the cellular environment that could affect the total intensity are likely affecting both alleles more or less equally, thus not masking the genetic effect on the relative intensity in chromatin state.

In the case of PCA of families 1 and 2 (Figure 2B) using the relative intensity, the largest variance, captured by PC1, was due to the difference between family 1 and family 2. In the case of PCA analysis of families 3 and 4 (Figure 3, top right), the largest variance (always captured by PC1 because of the algorithm) was due to the difference between control and antibodies targeting active chromatin states (H3Ac and H3K4). PC2 was the vector that contained the second largest variance in these data, corresponding to the difference between family 3 and family 4. The conclusion of genetic influence on chromatin state is supported by the clustering of the families when samples are projected in the 2-D space defined by PC1 and PC2. The conclusion is valid regardless of whether the separation is on PC1 or PC2, which is determined by the variance-covariance structure of the data. In other words, principal components are data driven. PCA is an unsupervised method. Furthermore, our allelic segregation analysis agrees very well with Mendelian inheritance of the chromatin state (Figures 5 and S8), thus providing direct support for genetic influence on the chromatin state.

It is interesting to note that familial aggregation of allelic-specific DNA methylation variation at imprinted gene loci has been previously reported [19] as well as Mendelian inheritance of DNA methylation [20]. A total of three recent studies also indicated germline inheritance of methylation epimutation in *MSH2* and *MLH1* in families with hereditary nonpolyposis colorectal cancer [21–23]. DNA methylation and histone acetylation showed nearly identical patterns in young monozygotic twins but marked differences in old monozygotic twins [24]. All these observations support the notion of the influence of genetic inheritance on epigenetic processes. A genetic effect on chromatin state is well known in model organisms. Examples include position-effect variegation in Drosophila melanogaster [25]. A related observation is transgenerational epigenetic inheritance. For example, agouti viable yellow mice display inheritance of yellow fur as a result of incomplete erasure of the methylation signal associated with a retrotransposon insertion [16], and kinked-tail mice transmit phenotype through multiple generations due to the loss of the silent epigenetic state at the *Axin* gene [26] as well as a heritable white-tail phenotype associated with Kit-specific microRNAs [17]. Meiotic transmission of epigenetic states has also been described in several studies in plant [27–29].

Although total gene expression as expression quantitative trait loci was regulated by genetic loci [6–8], the detection of the expression quantitative trait loci usually required a much larger sample size than the 12 samples used here. Our ability to detect familial aggregation by allele-specific chromatin state, but not in total chromatin state, resulted from the increased specificity of probing chromatin state with the relative intensity (A/A + B). The use of PCA might further enhance our ability to detect the genetic effects on chromatin state, since PCA had the capacity to detect a robust signal captured in the principal components even though signals from individual SNPs might be weak. The allelic differences in chromatin provided an explanation for the observed allelic variation in gene expression [5].

## Materials and Methods

### ChIP.

ChIP was carried out using a ChIP assay kit (Upstate, (http://www.upstate.com/img/coa/17-295-33519A.pdf). Lymphoblastoid cells of 24 individuals from CEPH/Utah pedigrees (family identification 1347, 1362, 1331, and 1413) were used in this study. ChIP was carried out using a ChIP assay kit (Upstate) with antibodies against histone H3 acetylated at K9 and K14 (Upstate, 06–599), dimethylated at K4 (Upstate, 07–030), dimethylated at K9 (Upstate, 07–441), dimethylated at K27 (Upstate, 07–452), trimethylated at K27 (Upstate, 07–449), and Pol II (Abcam, ab5408, http://www.abcam.com). All cell lines are described in Table S3. Briefly, 2 × 10^7^ cells were grown in RPMI medium 1,640 supplemented with 2 mM L-glutamine and 15% FBS. The cells were fixed by adding formaldehyde solution into the culture medium to a final concentration of 1%. After centrifugation the cell pellets were rinsed twice with an ice-cold PBS solution and then suspended in a lysis buffer (all buffers used in the ChIP experiment are described in http://www.upstate.com/img/coa/17-295-33519A.pdf). Sonication was performed on ice using Cole Parmer economical ultrasonic processor at power 9 for 12 cycles of sonication, each cycle for 10 s followed by a 30-s break on ice. The cell pellets were centrifuged at 10,000 RCF (×g) for 10 min, and the resulting lysates in the supernatant were stored at −80 °C until use. The chromatin lysates were diluted by 10-fold in a ChIP dilution buffer. They were precleared by Salmon sperm protein A agarose and incubated with each of the six antibodies individually overnight at 4 °C. The chromatin complexes were sequentially washed in low salt, high salt, LiCl salt, and TE buffers. The protein/DNA complex was eluted in an SDS elution buffer (1% SDS, 0.1 M NaHCO3). The crosslink between protein and DNA was reversed. The protein/DNA complex was treated with Proteinase K. DNAs were purified using Qiagen mini-elute reaction clean-up kit (http://www1.qiagen.com).

### HaploChIP assay using OLA.

PCR was carried out using primer pairs described in Table S2. Antarctic Phosphatase (New England Biolabs, http://www.neb.com) and Exonuclease I (New England Biolabs) was used to remove unincorporated primers and dNTPs. Allele-specific OLA was carried out in a 5-μl reaction containing 1× Ampligase buffer (Epicentre Biotechnologies, http://www.epibio.com), 100 nM each ligation primers, 0.5 U Ampligase, and 1 μl of phospho/exo treated PCR product (∼10 ng) for 30 cycles, with each cycle at 95 °C for 30 s, 50 °C for 30 s, and 65 °C for 2 min. All primers are described in Table S2. Ligation products were resolved by ABI3730XL genetic analyzer and analyzed using GeneMapper 3.5 software (Applied Biosystems, http://www.appliedbiosystems.com).

### HaploChIP-on-Chip assay using the 10K SNP ChIP.

We treated 500-ng input DNA or 50-ng immunoprecipitated DNAs in the ChIP experiment with mung bean nuclease to flush the ends. The DNA was phosphorylated and ligated to an Xba-linker (Table S2). Following an Xba I digestion, DNA was purified by Qiagen mini-elute reaction clean-up kit and was ligated to an Xba-adaptor. DNA was then amplified using an Xba-primer. This amplification step did not introduce biased representation of the initial ChIP DNA (Figure S7). It also retained the allelic specificity as demonstrated by the experiment described in Figures 1A and S6. Next, 10-μg PCR products from the input or 5-μg PCR products from the ChIP experiments were digested and labeled as described in the 10K SNP chip manual. We carried out the hybridization, washing, and scanning as described in the manual.

### Statistical analyses.

All statistical analyses were developed using R and Splus packages. The missing values in PM or MM probes were replaced by the mean MM across all SNPs. For each SNP, we computed the ratio PM/MM and then applied the Robust Multi-array Average (RMA) method [30]. Probe intensity was computed by the function of max(mean(log2[PM/MM], 0) for allele A and allele B. The intensity at the probe set level was the average of the ten pairs of the probes from each allele of the SNP. The signal for each allele of an SNP was evaluated by t-test for the measurement of (PM − MM) with H0 hypothesis of (PM − MM) = 0 for the ten probes for a given SNP. We chose a *p*-value of 0.01 as a threshold for the presence of a signal.

We used PCA to visualize similarity and variability among the 96 samples containing the ChIP data done on 12 individuals, each characterized with six antibodies plus the controls of input and DNA, using either the total binding intensity (A + B) or the relative binding intensity (A/A + B). PCA transforms the data matrix by rotating the coordinate system. After transformation, we had a new set of variables, denoted by principal components. Each principal component was a linear combination of the original variables with different weights (loadings). The loadings reflected the degree of contribution of each SNP to the principal component. PCA has two useful mathematic properties. First, all principal components are orthogonal to each other so the total variance is the sum of variances from each principal component. Second, principal components are ranked in such a way so that PC1 accounts for the largest variance in the data followed by PC2. In our data, the first two principal components typically accounted for about 10%–50% of the total variance. Therefore, we focused our analyses in two dimensions instead of the original 10,000 dimensions. The utilities of the PCA in this study are 2-fold. First, PCA provides dimension reduction, allowing visualization of data structure in 2-D. Second, it provides a quantitative assessment of data structure and interactions among variables. In this study, the data structure refers to the clustering of samples by family or antibody type. The separation of samples by different principal components reflects the degree of difference due to either family or antibody. The separation in PC1 is always the largest, by definition, due to the PCA algorithm. The relative contribution of the components can be assessed by eigen-values, which are provided in Tables S4 and S5.

The expected value of relative binding intensity (A/A + B) is 0.5. Deviation from 0.5 for an SNP among heterozygous individuals for genomic DNA and input suggests erroneous behavior of the SNP. We removed SNPs whose deviation from 0.5 exceeded two standard deviations. A total of 2,365 SNPs were removed by this criterion. We used 0.5 for homozygous individuals in the PCA for the relative binding intensity (A/A + B). All samples were projected in the space defined by the first and second principal components.

## Supporting Information

Figure S1 PCA Was Performed with the Term (Ia + Ib) for the Samples in CEPH Family 1347 and 1362 Clustering of the samples by antibody with total chromatin-binding activity was observed. This is similar to Figure 2A except for the labeling and inclusion of ChIP experiment using antibody targeting MECP2 protein in Figure S1. We include complete information about cell lines and antibodies. The samples are described in Table S3.
Color codings: genomic DNA, black; input, green; proteins associated with active chromatin, red; and proteins associated with inactive chromatin, blue.
g, genomic DNA; i, input nuclear extract; Ac, histone H3 lysine 9/14 acetylation; K4, histone H3 lysine 4 dimethylation; Pol, Pol II; K9, histone H3 lysine 9 dimethylation; K27d, histone H3 lysine 27 dimethylation; K27t, histone H3 lysine 27 trimethylation; and mecp, MECP2.
(9 KB PDF)

Figure S2 PCA Was Performed with the Ratio Ia/(Ia + Ib) for Samples in CEPH Family 1347 and 1362 Clustering of the samples by family with allelic chromatin-binding activity was observed. The sample and antibody labeling are the same as Figure S1. This is similar to Figure 2B except for the labeling and inclusion of MECP2.
(9 KB PDF)

Figure S3 PCA Was Performed with the Term (Ia + Ib) for the Samples in CEPH Family 1331 and 1413 Clustering of the samples by antibody with total chromatin-binding activity was observed. This is identical to the top left figure in Figure 3 except for the labeling. The samples are described in Table S3.
Color codings: genomic DNA, black; input, green; and Ac, red.
g, genomic DNA; i, input DNA; and Ac, histone H3 lysine 9/14 acetylation.
(5 KB PDF)

Figure S4 PCA Was Performed with the Ratio Ia/(Ia + Ib) for the Samples in CEPH Family 1331 and 1413 Clustering of the samples by family with allelic chromatin-binding activity was observed. The sample and antibody labeling are the same as Figure S3. This is identical to the top right figure in Figure 3 except for the labeling.
Color codings: genomic DNA, black; input, green; and Ac, red.
(5 KB PDF)

Figure S5 PCA Was Performed with the Ratio Ia/(Ia + Ib) for the Samples in CEPH Family 1331 and 1413 Clustering of the samples by family with allelic chromatin binding activity was observed. The sample and antibody labeling are the same as Figure S4. This is similar to Figure S4 except for the omission of genomic DNAs in the PCA analysis.
(4 KB PDF)

Figure S6 Allelic Enrichment Analysis of ChIP Signal at *HPRT1, PGK1,* and *SNRPN* Gene Promoter by OLA Two peaks represent G allele (left) or T allele (right) for rs6634990 *(HPRT1),* G allele (left), or A allele (right) for rs2076628 *(PGK1),* and C allele (left) or T allele (right) for rs220030 *(SNRPN)*. Samples were from two CEPH families (identification 1347 and 1362). The samples are described in Table S3. All heterozygous samples were used for *SNRPN*. However, for X-linked *HPRT1* and *PGK1*, only female heterozygous samples without mosaic pattern of x-inactivation were used in this study.
Input, input DNA; Pol, RNA polymerase II; Ac, H3Ac; K4, H3K4.
(814 KB PDF)

Figure S7 Antibody Specificity and Amplification Specificity in ChIP-on-ChIP Experiment Were Evaluated by Quantitative Real-Time PCR Occupancies of Pol II or histone H3 modifications at six different loci were analyzed by quantitative PCR. ChIP DNAs or amplified ChIP DNAs from GM10859 were used as a template in quantitative PCR experiment to assess the signal enrichment at three active and three inactive loci. The genes analyzed here are *RPLP1*, *CD19*, and *GAPDH* for active regions, and *MYOD1*, *NES*, and *CD3G* for inactive regions. PCR was carried out in triplicate in a 10-μl volume with 250 nM forward and reverse primers using Power SYBR Green PCR Mastermix (Applied Biosystems), with ABI Prism 7900HT sequence detection system. PCR cycling condition was 50 °C for 2 min, 95 °C for 15 s, and 60 °C for 1 min for 40 cycles. Dilutions of GM10859 gDNA with predetermined concentrations and experimentally determined critical thresholds (CT) were used to construct a standard curve for calibrating the amount of target amplification. The amounts of target amplifications on the y-axis are expressed as a number of fold differences relative to either the input DNA (for the ChIP samples before amplification) or to the amplified input DNA (for the ChIP samples after amplification).
Pol, RNA polymerase II; Ac, histone H3 lysine 9/14 acetylation; K4, histone H3 lysine 4 dimethylation; K9, histone H3 lysine 9 dimethylation; K27di, histone H3 lysine 27 dimethylation; K27tri, histone H3 lysine 27 trimethylation; and Amp, amplified DNA. Data are represented as mean +/− standard deviation of triplicated sample.
(702 KB PDF)

Figure S8 Mendelian Inheritance Analysis of Allelic Specific Histone H3 Acetylation State Pedigree analysis was carried out for the SNPs shown in Figure 4. The results for four genes, rs938335 *(TMEM16D),* rs1414503 *(PKHD1),* rs590944 *(TCBA1),* and rs2346824 *(SYT9)* are shown in Figure 5, and the rest of the three genes, rs2409411 *(TIAM1),* rs270015 *(c6orf190),* and rs719535 *(ASTN2)* are shown in Figure S8. Each individual is shown with CEPH family identification, sample identification, and genetic information (SNP genotype or haplotype). The haplotype with higher chromatin binding activity (2 standard deviation above mean) is highlighted with red, whereas the haplotype with lower chromatin binding activity (2 standard deviation below mean) is highlighted with blue. Allelic fraction values (A/[A + B]) are also shown for heterozygous samples (>0.5 for A > B, 0.5 for A = B, and <0.5 for A < B). Filled circles or squares indicate the affected individual (allelic fraction is significantly different from 0.5), and a dot in a circle or square indicates a carrier of high or low allele. Genotype calls were all derived from gDNA call on 10K SNP chip and A or B alleles are assigned in alphabetical order of the nucleotides (A, C, G, T) for each SNP as defined by Affymetrix calling algorithm.
(698 KB PDF)

Table S1 Summary of All ChIP-on-chip Experiments Each row is one microarray experiment. There are eight microarray data for each sample in CEPH family 1362 and 1347. They are: gDNA, genomic DNA; input, input DNA; H3Ac, histone H3 lysine 9/14 acetylation; H3K4, histone H3 lysine 4 dimethylation; H3K9, histone H3 lysine 9 dimethylation; H3K27di, histone H3 lysine 27 dimethylation; H3K27tri, histone H3 lysine 27 trimethylation; and Pol, RNA polymerase II. We performed a subset of the ChIP-on-chip studies for samples in CEPH family 1331 and 1413. These are gDNA, input, H3Ac, and H3K4. The numbers in the SNPs column contain the numbers of SNPs with 10,204 corresponding to version 2 chip and 11,555 corresponding to version 1 chip. The numbers in the gDNA and input column contain a number of SNPs that were called in both genomic DNA and input samples. The percent call (genotype) and percent signal were from Affymetrix DAS software. The percent concordance has the percentage of identical calls between the calls made from genomic DNA and input among the SNPs listed in the gDNA and put column. Additional information for the samples can be found in Table S3.
(28 KB PDF)

Table S2 Oligos Used in Quantitative PCR, OLA, and ChIP-on-chip Experiment The phosphorylated Xba linker was formed by self-annealing of the oligo.
(27 KB PDF)

Table S3 Samples Used in ChIP-on-chip Experiment Additional information can be found at http://locus.umdnj.edu/nigms/ceph/ceph.html.
(25 KB PDF)

Table S4 The Eigen Values for PCA Shown in Figure 2
The sum of all eigen values is scaled to 1.
(20 KB PDF)

Table S5 The Eigen Values for PCA Shown in Figure 3
The sum of all eigen values is scaled to 1.
(18 KB PDF)

Table S6 Description of Samples Used in ChIP-on-chip Experiment Additional information can be found at http://locus.umdnj.edu/nigms/ceph/ceph.html.
(34 KB PDF)

Table S7 Demographic Information of the Samples Used in ChIP-on-chip Experiment Additional information can be found at http://locus.umdnj.edu/nigms/ceph/ceph.html.
(25 KB PDF)

Table S8 Minimum Information about a Microarray Experiment (MIAME) Checklist (140 KB DOC)

### Accession Numbers

The National Center for Biotechnology Information (NCBI) Entrez Gene (http://www.ncbi.nlm.nih.gov/entrez/query.fcgi?db=gene) accession numbers for the genes discussed in this paper are *ASTN2*, 23245; *C6orf190*, 387357; *CD19*, 930; *CD3G*, 917; *GAPDH*, 2597; *MYOD1*, 4654; *NES*, 10763; *PKHD1*, 5314; *RPLP1,* 6176; *SYT9*, 143425; *TCBA1*, 154215; *TIAM1*, 7074; and *TMEM16D*, 121601.

## Abbreviations

Ac: acetylated histone 3
CEPH: Centre d'Etude du Polymorphisme Humain
ChIP: chromatin immunoprecipitation
H3Ac: histone H3 lysine 9/14 acetylation
H3K4: histone H3 lysine 4 dimethylation
H3K9: histone H3 lysine 9 dimethylation
K27di: histone H3 lysine 27 dimethylation
K27tri: histone H3 lysine 27 trimethylation
OLA: oligo ligation assay
PC1: first principal component
PC2: second principal component
PCA: principal components analysis
Pol II: RNA polymerase II
SNP: single nucleotide polymorphism
