# Supplementary material for: Allele-Specific Chromatin Immunoprecipitation Studies Show Genetic Influence on Chromatin State in Human Genome
Source: PLoS Genet. 2007 May 18;3(5):e81. doi: 10.1371/journal.pgen.0030081 (PMC1868950; doi:10.1371/journal.pgen.0030081)
Supplement: Supplementary file 1 [file pgen.0030081.sg001.pdf]

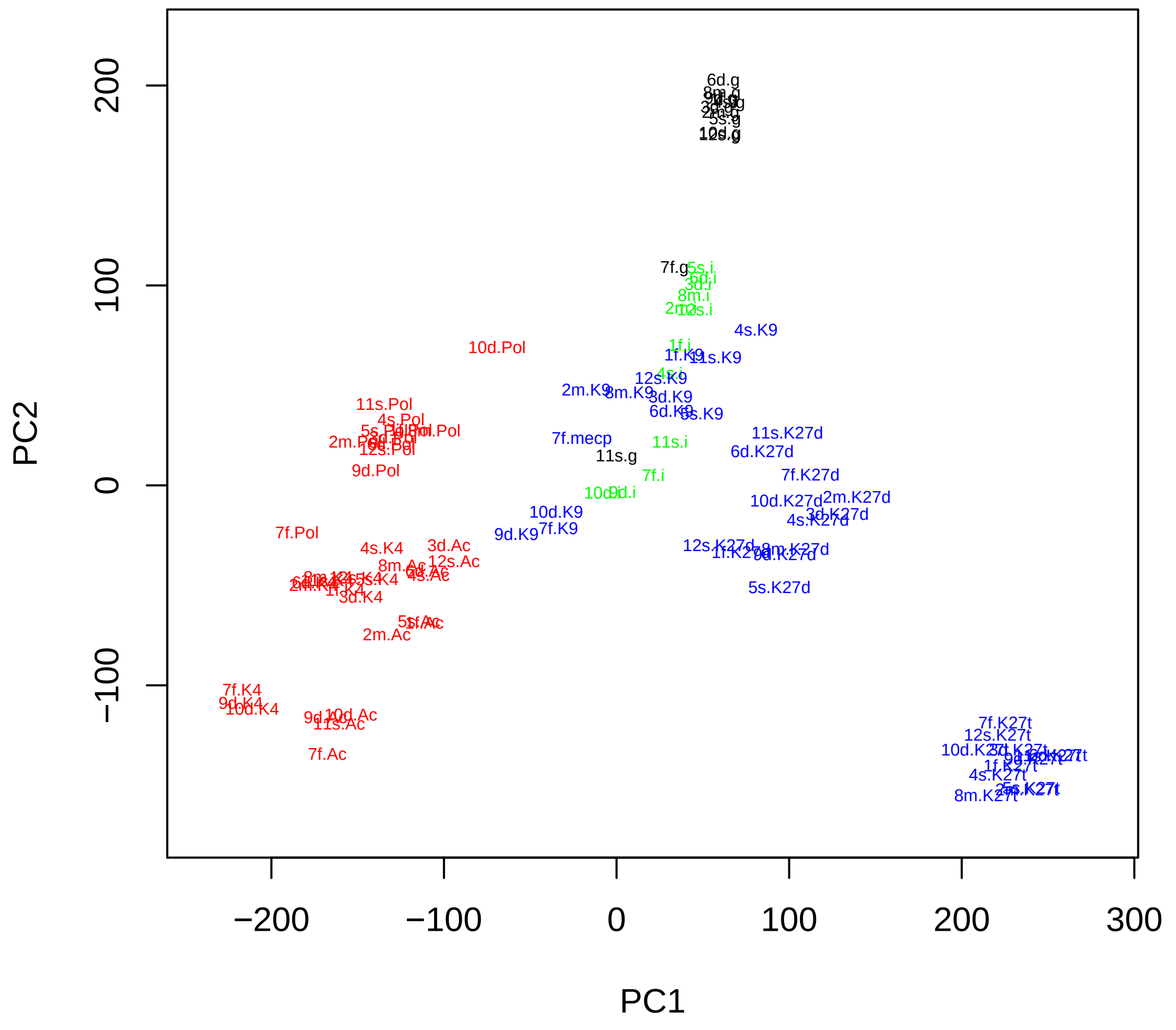
PC1
PC2
−200
−100
0
100
200
300
200
100
0
−100
6d.g
10d.g
7f.g
11s.g
5s.i
8m.i
1f.i
11s.i
7f.i
4s.K9
11s.K9
12s.K9
2m.K9
8m.K9
3d.K9
6d.K9
5s.K9
1f.K9
7f.mecp
10d.K9
7f.K9
9d.K9
11s.K27d
6d.K27d
7f.K27d
10d.K27d
2m.K27d
3d.K27d
4s.K27d
12s.K27d
9d.K27d
5s.K27d
7f.K27t
12s.K27t
4s.K27t
8m.K27t
10d.Pol
11s.Pol
4s.Pol
12s.Pol
9d.Pol
7f.Pol
4s.K4
3d.K4
3d.Ac
12s.Ac
8m.Ac
4s.Ac
2m.Ac
7f.K4
10d.K4
10d.Ac
11s.Ac
7f.Ac
