# Supplementary material for: Allele-Specific Chromatin Immunoprecipitation Studies Show Genetic Influence on Chromatin State in Human Genome
Source: PLoS Genet. 2007 May 18;3(5):e81. doi: 10.1371/journal.pgen.0030081 (PMC1868950; doi:10.1371/journal.pgen.0030081)
Supplement: Supplementary file 2 [file pgen.0030081.sg002.pdf]

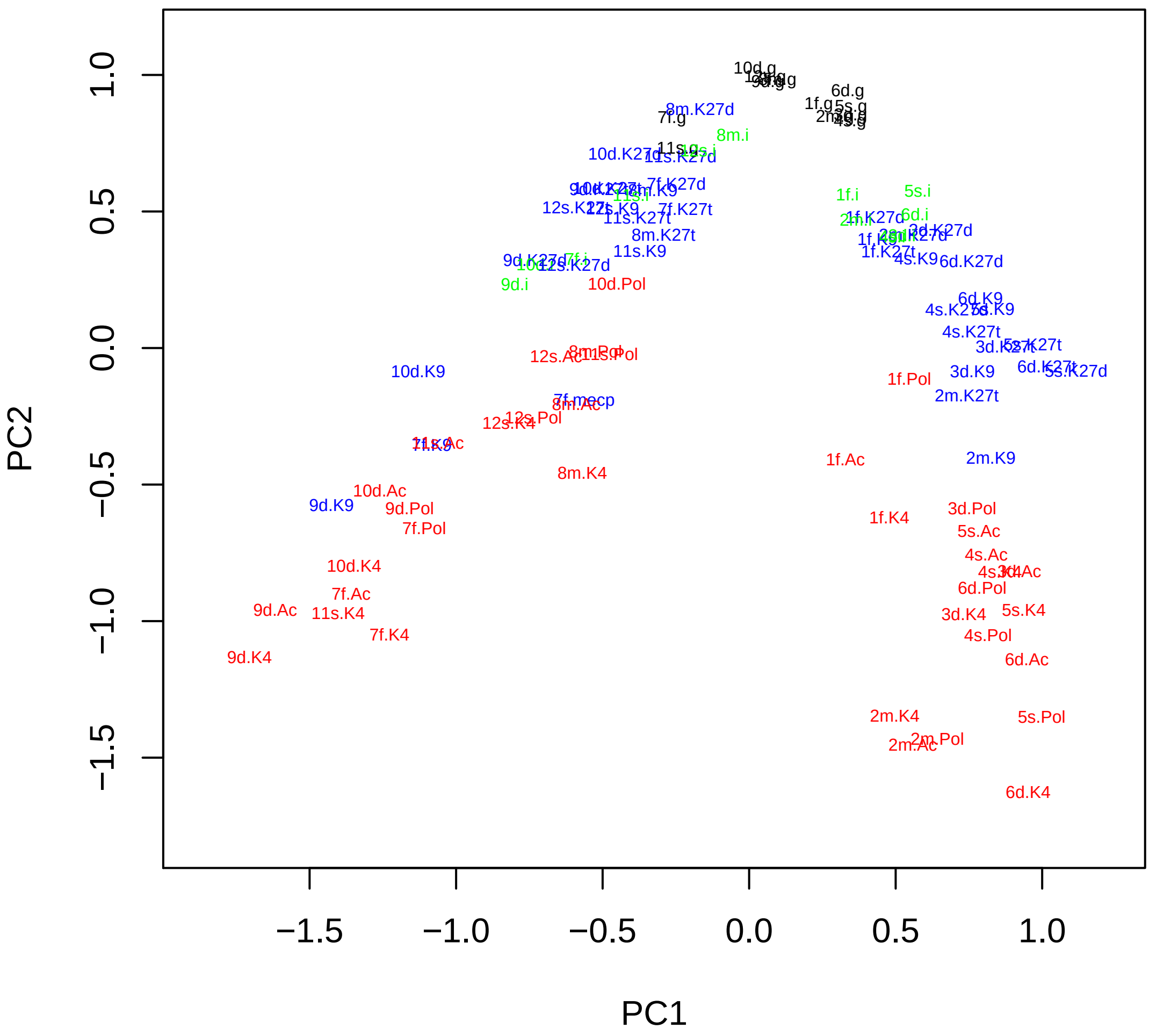
PC1
PC2
−1.5
−1.0
−0.5
0.0
0.5
1.0
10d.g
6d.g
1f.g
5s.g
4s.g
7f.g
8m.K27d
8m.i
10d.K27d
11s.K27d
7f.K27d
7f.K27t
12s.K27t
11s.K27t
8m.K27t
11s.K9
1f.i
5s.i
6d.i
2m.i
1f.K27d
3d.K27d
1f.K27t
4s.K9
6d.K27d
9d.K27d
12s.K27d
9d.i
10d.Pol
6d.K9
5s.K9
4s.K27t
3d.K27t
5s.K27t
6d.K27t
5s.K27d
3d.K9
2m.K27t
1f.Pol
12s.Ac
11s.Pol
10d.K9
7f.mecp
8m.Ac
12s.Pol
12s.K4
11s.Ac
7f.K9
8m.K4
1f.Ac
2m.K9
10d.Ac
9d.K9
9d.Pol
7f.Pol
1f.K4
3d.Pol
5s.Ac
4s.Ac
3d.Ac
6d.Pol
10d.K4
7f.Ac
9d.Ac
11s.K4
7f.K4
3d.K4
5s.K4
4s.Pol
6d.Ac
9d.K4
2m.K4
5s.Pol
2m.Pol
2m.Ac
6d.K4
