# Supplementary material for: Allele-Specific Chromatin Immunoprecipitation Studies Show Genetic Influence on Chromatin State in Human Genome
Source: PLoS Genet. 2007 May 18;3(5):e81. doi: 10.1371/journal.pgen.0030081 (PMC1868950; doi:10.1371/journal.pgen.0030081)
Supplement: Supplementary file 3 [file pgen.0030081.sg003.pdf]

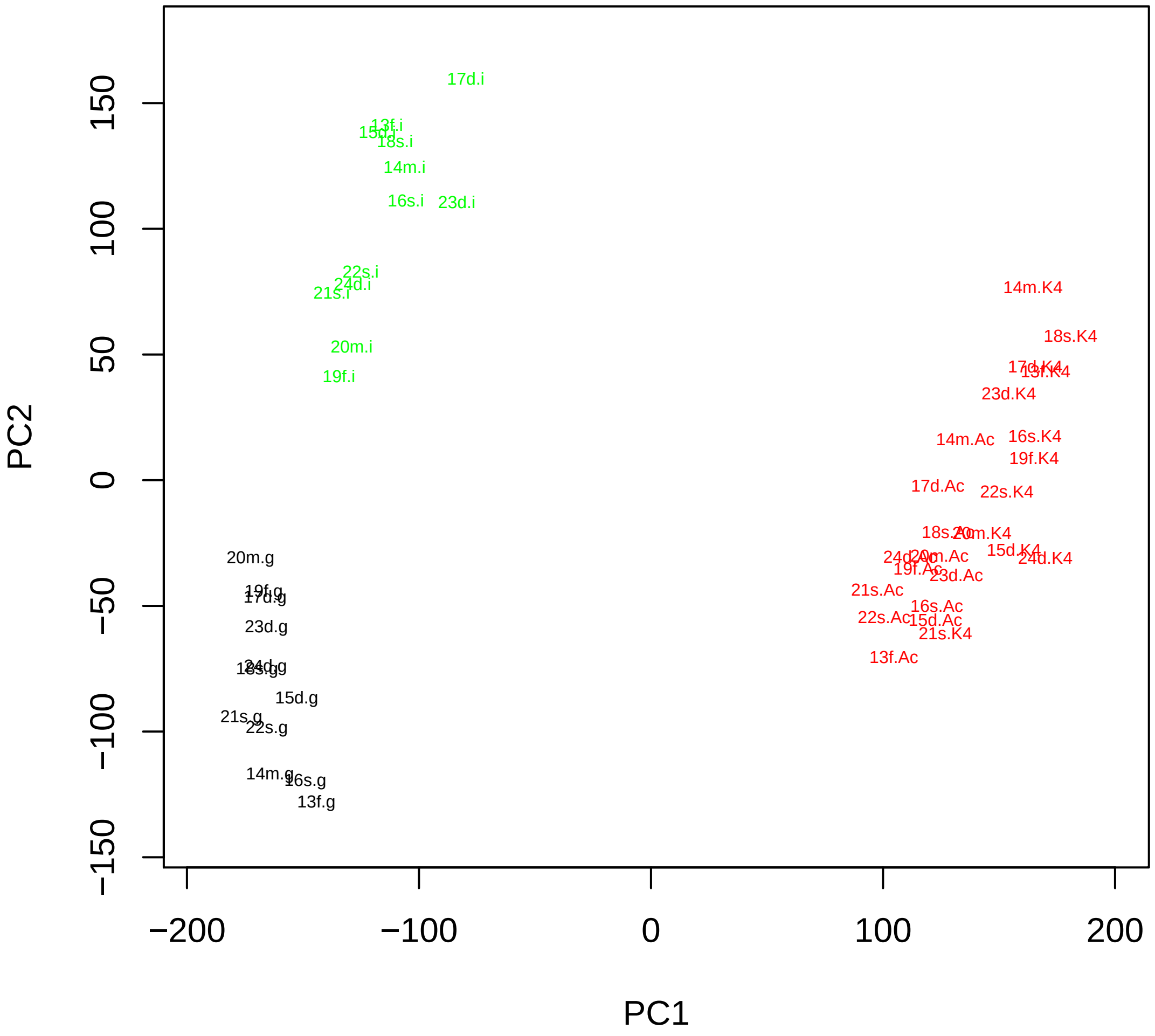
PC1
PC2
−200
−100
0
100
200
−150
−100
−50
0
50
100
150
17d.i
13f.i
15d.i
18s.i
14m.i
16s.i
23d.i
22s.i
24d.i
21s.i
20m.i
19f.i
20m.g
19f.g
17d.g
23d.g
24d.g
18s.g
15d.g
21s.g
22s.g
14m.g
16s.g
13f.g
14m.K4
18s.K4
17d.K4
13f.K4
23d.K4
14m.Ac
16s.K4
19f.K4
17d.Ac
22s.K4
18s.Ac
20m.K4
15d.K4
24d.K4
24d.Ac
20m.Ac
19f.Ac
23d.Ac
21s.Ac
16s.Ac
22s.Ac
15d.Ac
21s.K4
13f.Ac
