# Supplementary material for: Allele-Specific Chromatin Immunoprecipitation Studies Show Genetic Influence on Chromatin State in Human Genome
Source: PLoS Genet. 2007 May 18;3(5):e81. doi: 10.1371/journal.pgen.0030081 (PMC1868950; doi:10.1371/journal.pgen.0030081)
Supplement: Supplementary file 5 [file pgen.0030081.sg005.pdf]

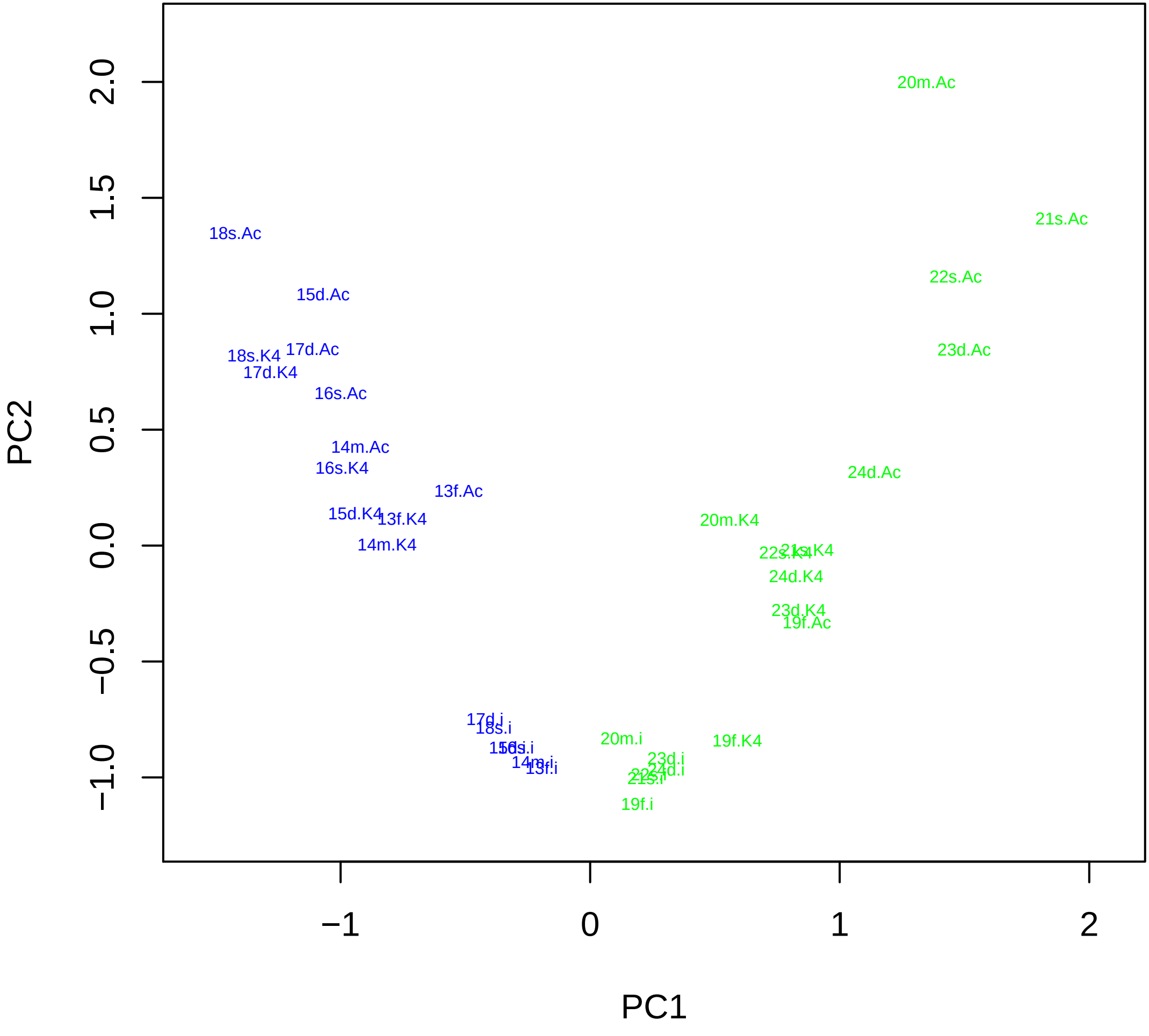

PC1
PC2
−1
0
1
2
−1.0
−0.5
0.0
0.5
1.0
1.5
2.0
18s.Ac
15d.Ac
18s.K4
17d.Ac
17d.K4
16s.Ac
14m.Ac
16s.K4
13f.Ac
15d.K4
13f.K4
14m.K4
17d.i
18s.i
14m.i
13f.i
20m.Ac
21s.Ac
22s.Ac
23d.Ac
24d.Ac
20m.K4
24d.K4
23d.K4
19f.Ac
20m.i
19f.K4
23d.i
24d.i
19f.i
