# Supplementary material for: Allele-Specific Chromatin Immunoprecipitation Studies Show Genetic Influence on Chromatin State in Human Genome
Source: PLoS Genet. 2007 May 18;3(5):e81. doi: 10.1371/journal.pgen.0030081 (PMC1868950; doi:10.1371/journal.pgen.0030081)
Supplement: Supplementary file 6 [file pgen.0030081.sg006.pdf]

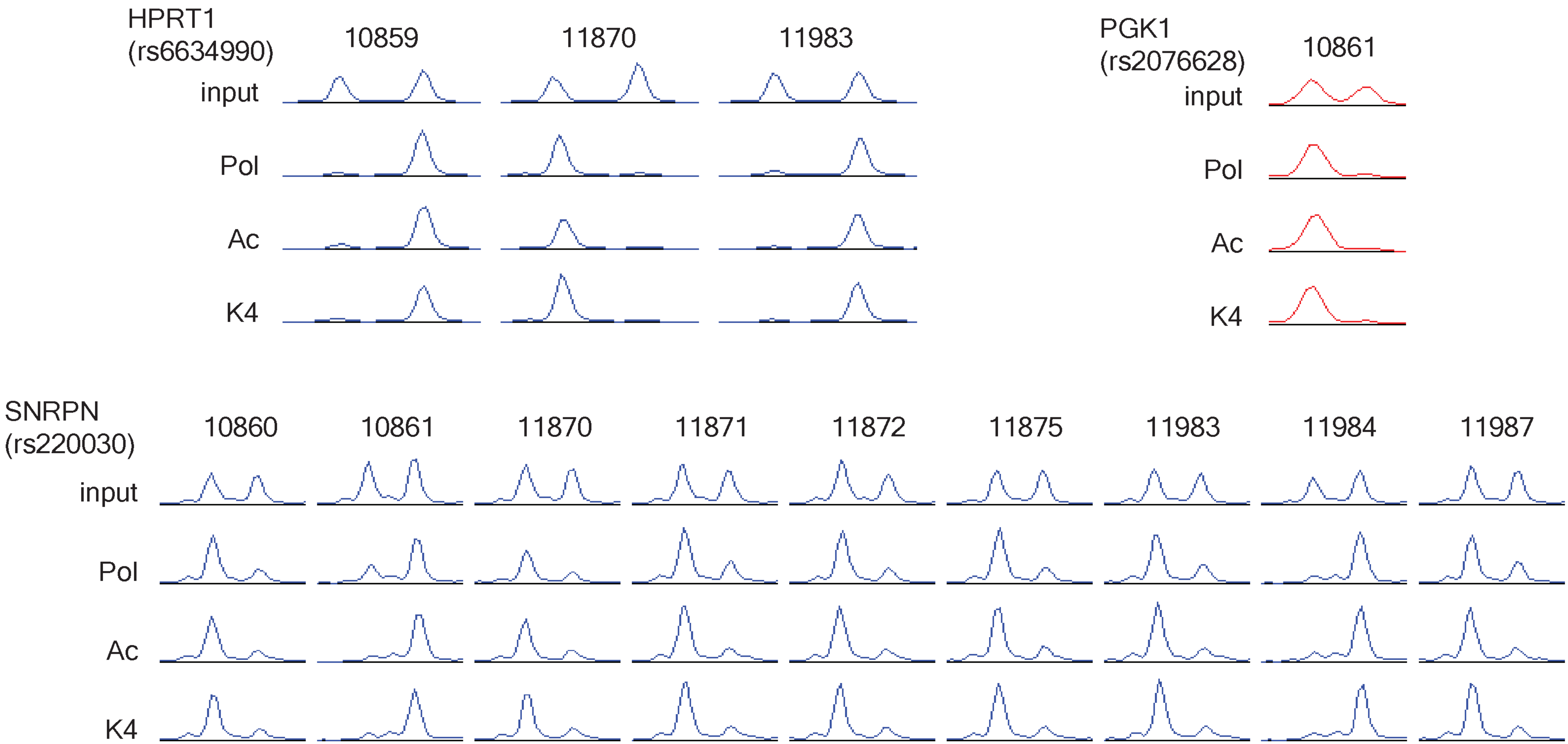
HPRT1
(rs6634990)
10859
11870
11983
input
Pol
Ac
K4
PGK1
(rs2076628)
10861
input
Pol
Ac
K4
SNRPN
(rs220030)
10860
10861
11870
11871
11872
11875
11983
11984
11987
input
Pol
Ac
K4
