# Supplementary material for: Allele-Specific Chromatin Immunoprecipitation Studies Show Genetic Influence on Chromatin State in Human Genome
Source: PLoS Genet. 2007 May 18;3(5):e81. doi: 10.1371/journal.pgen.0030081 (PMC1868950; doi:10.1371/journal.pgen.0030081)
Supplement: Supplementary file 7 [file pgen.0030081.sg007.pdf]

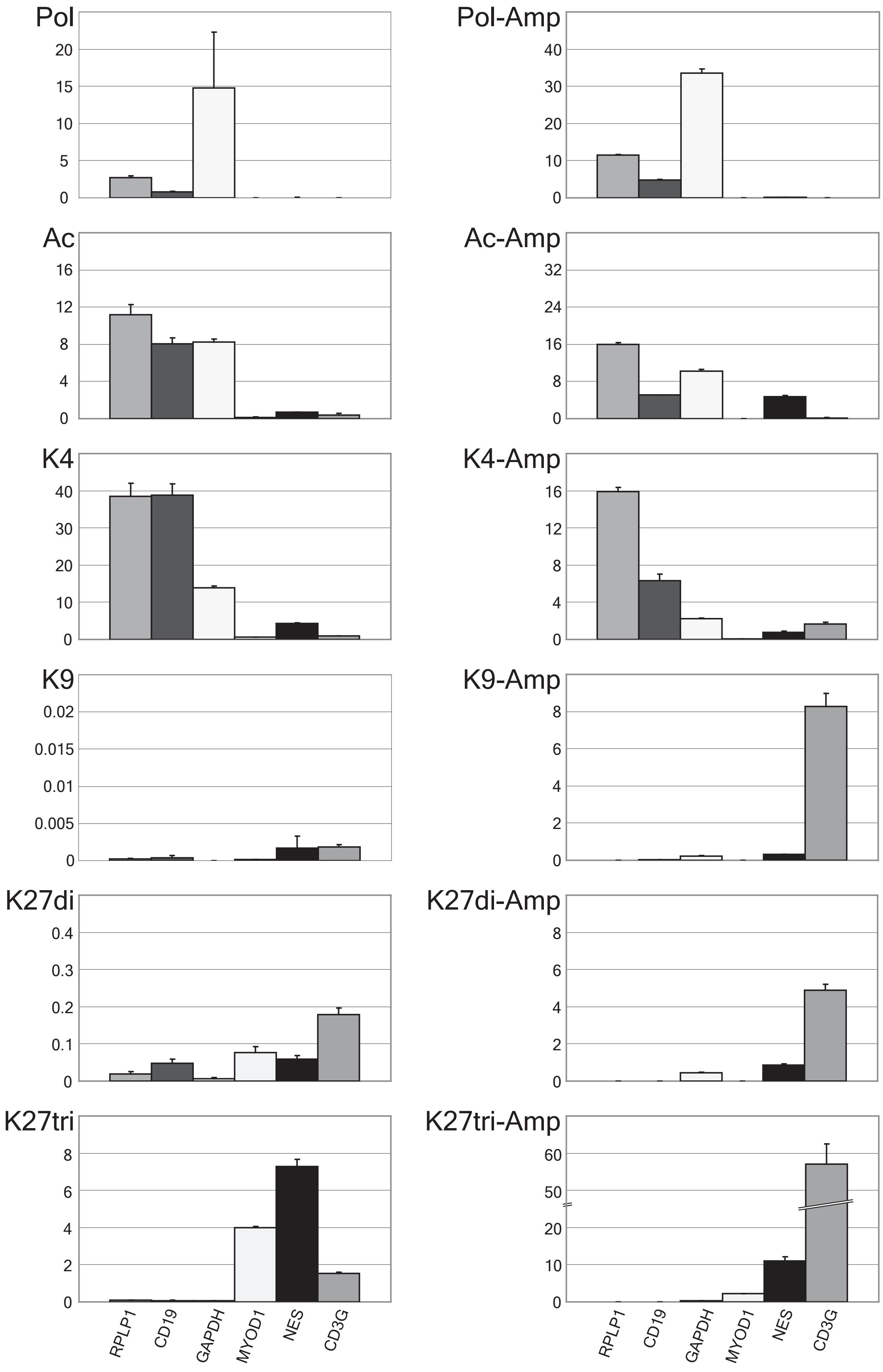

Pol
Pol-Amp
Ac
Ac-Amp
K4
K4-Amp
K9
K9-Amp
K27di
K27di-Amp
K27tri
K27tri-Amp
RPLP1
CD19
GAPDH
MYOD1
NES
CD3G
