# Supplementary material for: Allele-Specific Chromatin Immunoprecipitation Studies Show Genetic Influence on Chromatin State in Human Genome
Source: PLoS Genet. 2007 May 18;3(5):e81. doi: 10.1371/journal.pgen.0030081 (PMC1868950; doi:10.1371/journal.pgen.0030081)
Supplement: Supplementary file 8 [file pgen.0030081.sg008.pdf]

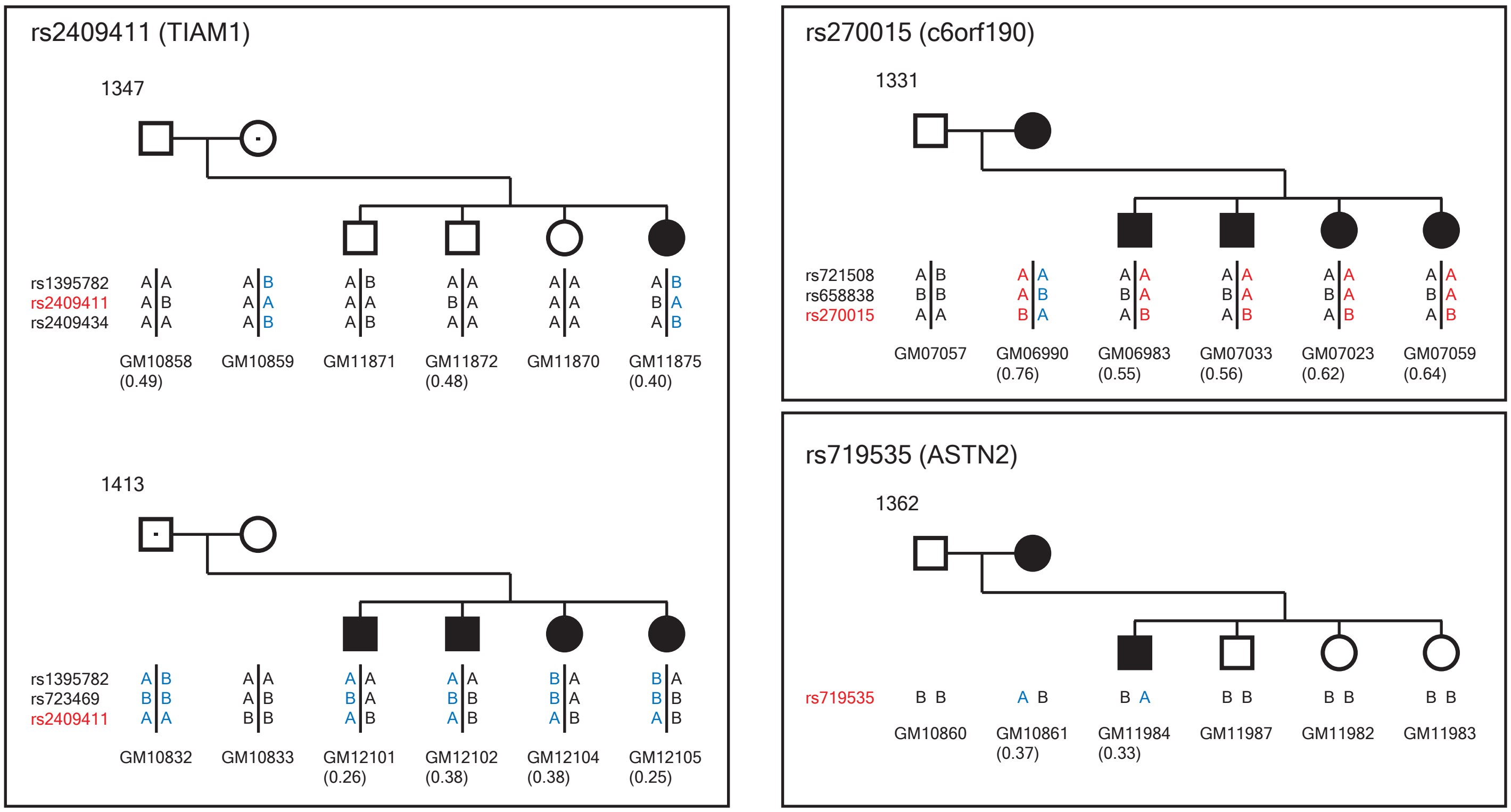

rs2409411 (TIAM1)
1347
rs1395782
rs2409411
rs2409434
A A
A B
A A
A B
A A
A B
A B
A A
A B
A A
B A
A A
A A
A A
A A
A B
B A
A B
GM10858
(0.49)
GM10859
GM11871
GM11872
(0.48)
GM11870
GM11875
(0.40)
1413
rs1395782
rs723469
rs2409411
A B
B B
A A
A A
A B
B B
A A
B A
A B
A A
B B
A B
B A
B A
A B
B A
B B
A B
GM10832
GM10833
GM12101
(0.26)
GM12102
(0.38)
GM12104
(0.38)
GM12105
(0.25)
rs270015 (c6orf190)
1331
rs721508
rs658838
rs270015
A B
B B
A A
A A
A B
B A
A A
B A
A B
A A
B A
A B
A A
B A
A B
A A
B A
A B
GM07057
GM06990
(0.76)
GM06983
(0.55)
GM07033
(0.56)
GM07023
(0.62)
GM07059
(0.64)
rs719535 (ASTN2)
1362
rs719535
B B
A B
B A
B B
B B
B B
GM10860
GM10861
(0.37)
GM11984
(0.33)
GM11987
GM11982
GM11983
