# Supplementary material for: Allele-Specific Chromatin Immunoprecipitation Studies Show Genetic Influence on Chromatin State in Human Genome
Source: PLoS Genet. 2007 May 18;3(5):e81. doi: 10.1371/journal.pgen.0030081 (PMC1868950; doi:10.1371/journal.pgen.0030081)
Supplement: Supplementary file 9 [file pgen.0030081.st001.pdf]

sample	antibody	version	SNPs	percent call	percent signal	gDNA and input	percent concordance
GM06983	gDNA	2	10204	98.96	99.94		
GM06990	gDNA	2	10204	98.82	99.91		
GM07023	gDNA	2	10204	98.52	99.95		
GM07033	gDNA	2	10204	98.28	99.83		
GM07057	gDNA	2	10204	97.70	99.75		
GM07059	gDNA	2	10204	98.85	99.94		
GM10832	gDNA	2	10204	98.93	99.92		
GM10833	gDNA	2	10204	98.18	99.96		
GM10858	gDNA	2	10204	99.27	99.95		
GM10859	gDNA	2	10204	99.10	99.93		
GM10860	gDNA	1	11555	96.66	99.27		
GM10861	gDNA	2	10204	99.27	99.93		
GM11870	gDNA	2	10204	98.36	99.91		
GM11871	gDNA	2	10204	98.49	99.92		
GM11872	gDNA	2	10204	98.90	99.97		
GM11875	gDNA	2	10204	99.32	99.99		
GM11982	gDNA	2	10204	98.61	99.33		
GM11983	gDNA	2	10204	98.83	99.33		
GM11984	gDNA	1	11555	84.79	91.94		
GM11987	gDNA	2	10204	99.52	99.99		
GM12101	gDNA	2	10204	98.54	99.97		
GM12102	gDNA	2	10204	98.49	99.89		
GM12104	gDNA	2	10204	99.14	99.92		
GM12105	gDNA	2	10204	98.87	99.97		
GM06983	input	2	10204	87.24	95.78	8867	99.41
GM06990	input	2	10204	85.78	95.39	8703	99.44
GM07023	input	2	10204	88.47	96.76	8957	99.31
GM07033	input	2	10204	85.83	95.37	8674	99.37
GM07057	input	2	10204	87.53	96.06	8795	99.20
GM07059	input	2	10204	78.31	91.63	7956	98.79
GM10832	input	2	10204	90.87	98.25	9239	99.38
GM10833	input	2	10204	92.28	98.54	9347	99.73
GM10858	input	2	10204	79.76	92.26	8125	99.20
GM10859	input	2	10204	85.69	95.77	8719	99.39
GM10860	input	1	11555	73.36	86.23	8393	98.95
GM10861	input	2	10204	87.87	97.09	8931	99.41
GM11870	input	2	10204	81.81	95.00	8280	98.83
GM11871	input	2	10204	74.75	89.36	7561	98.97
GM11872	input	2	10204	88.86	96.63	9014	99.69
GM11875	input	2	10204	85.50	95.90	8700	99.54
GM11982	input	1	11555	64.01	82.03	6556	97.90
GM11983	input	1	11555	59.45	77.63	6085	97.01
GM11984	input	1	11555	80.74	92.06	8361	99.16
GM11987	input	2	10204	81.77	93.95	8334	98.85
GM12101	input	2	10204	91.91	98.24	9317	99.70
GM12102	input	2	10204	90.76	97.22	9206	99.78
GM12104	input	2	10204	80.37	91.60	8179	99.34
GM12105	input	2	10204	89.77	97.23	9111	99.59
GM06983	H3Ac	2	10204	40.49	61.56		

GM06990	H3Ac	2	10204	39.20	58.60
GM07023	H3Ac	2	10204	35.54	55.26
GM07033	H3Ac	2	10204	36.57	57.88
GM07057	H3Ac	2	10204	40.18	62.97
GM07059	H3Ac	2	10204	35.43	54.82
GM10832	H3Ac	2	10204	35.79	55.45
GM10833	H3Ac	2	10204	28.23	45.18
GM10858	H3Ac	2	10204	36.31	57.88
GM10859	H3Ac	2	10204	37.88	59.65
GM10860	H3Ac	1	11555	27.24	38.68
GM10861	H3Ac	2	10204	42.34	65.99
GM11870	H3Ac	2	10204	42.85	66.74
GM11871	H3Ac	2	10204	43.71	66.59
GM11872	H3Ac	2	10204	39.24	60.76
GM11875	H3Ac	2	10204	34.02	57.01
GM11982	H3Ac	1	11555	24.39	39.39
GM11983	H3Ac	1	11555	34.33	52.57
GM11984	H3Ac	1	11555	26.35	36.05
GM11987	H3Ac	2	10204	48.84	72.49
GM12101	H3Ac	2	10204	18.79	31.32
GM12102	H3Ac	2	10204	17.46	29.63
GM12104	H3Ac	2	10204	21.63	34.98
GM12105	H3Ac	2	10204	34.40	53.42
GM06983	H3K4	2	10204	51.16	69.63
GM06990	H3K4	2	10204	53.93	72.87
GM07023	H3K4	2	10204	58.02	78.44
GM07033	H3K4	2	10204	57.5	78.09
GM07057	H3K4	2	10204	52.59	72.7
GM07059	H3K4	2	10204	50.51	70.24
GM10832	H3K4	2	10204	59.2	78.4
GM10833	H3K4	2	10204	63.58	83.21
GM10858	H3K4	2	10204	32.35	52.82
GM10859	H3K4	2	10204	36.23	58.39
GM10860	H3K4	1	11555	19.12	29.21
GM10861	H3K4	2	10204	38.79	61.26
GM11870	H3K4	2	10204	33.32	53.80
GM11871	H3K4	2	10204	43.97	66.39
GM11872	H3K4	2	10204	39.16	60.82
GM11875	H3K4	2	10204	33.08	54.36
GM11982	H3K4	1	11555	26.98	42.14
GM11983	H3K4	1	11555	22.68	37.10
GM11984	H3K4	1	11555	22.22	38.98
GM11987	H3K4	2	10204	39.41	62.29
GM12101	H3K4	2	10204	63.63	83.97
GM12102	H3K4	2	10204	57.33	78.17
GM12104	H3K4	2	10204	58.54	77.27
GM12105	H3K4	2	10204	55.3	75.37
GM10858	H3K9	2	10204	62.80	83.29
GM10859	H3K9	2	10204	28.43	44.76
GM10860	H3K9	1	11555	35.42	49.92
GM10861	H3K9	2	10204	58.28	79.39

GM11870	H3K9	2	10204	54.43	77.76
GM11871	H3K9	2	10204	67.80	87.28
GM11872	H3K9	2	10204	54.48	76.40
GM11875	H3K9	2	10204	48.05	67.52
GM11982	H3K9	1	11555	28.03	46.45
GM11983	H3K9	1	11555	40.43	61.89
GM11984	H3K9	2	10204	63.58	84.10
GM11987	H3K9	2	10204	54.80	77.38
GM10858	H3K27di	2	10204	44.91	65.53
GM10859	H3K27di	2	10204	69.39	86.05
GM10860	H3K27di	2	10204	70.61	87.13
GM10861	H3K27di	2	10204	56.12	75.95
GM11870	H3K27di	2	10204	62.19	79.52
GM11871	H3K27di	2	10204	52.32	73.27
GM11872	H3K27di	2	10204	41.80	60.40
GM11875	H3K27di	2	10204	60.31	79.76
GM11982	H3K27di	2	10204	43.12	63.32
GM11983	H3K27di	2	10204	60.38	81.39
GM11984	H3K27di	2	10204	70.34	87.53
GM11987	H3K27di	2	10204	39.52	60.17
GM10858	H3K27tri	2	10204	57.14	73.88
GM10859	H3K27tri	2	10204	56.81	73.22
GM10860	H3K27tri	2	10204	60.11	76.81
GM10861	H3K27tri	2	10204	48.00	65.56
GM11870	H3K27tri	2	10204	53.39	70.78
GM11871	H3K27tri	2	10204	52.22	69.24
GM11872	H3K27tri	2	10204	52.18	68.01
GM11875	H3K27tri	2	10204	59.06	74.52
GM11982	H3K27tri	2	10204	59.62	76.06
GM11983	H3K27tri	2	10204	51.54	69.46
GM11984	H3K27tri	2	10204	57.22	73.95
GM11987	H3K27tri	2	10204	54.34	73.07
GM10858	Pol	2	10204	35.30	57.60
GM10859	Pol	2	10204	20.00	33.95
GM10860	Pol	1	11555	31.03	46.01
GM10861	Pol	2	10204	40.23	63.85
GM11870	Pol	2	10204	42.40	65.24
GM11871	Pol	2	10204	34.46	52.90
GM11872	Pol	2	10204	26.26	45.55
GM11875	Pol	2	10204	29.99	47.04
GM11982	Pol	2	10204	25.65	42.23
GM11983	Pol	2	10204	62.14	84.35
GM11984	Pol	2	10204	38.48	60.53
GM11987	Pol	2	10204	27.24	45.23
