# Supplementary material for: Allele-Specific Chromatin Immunoprecipitation Studies Show Genetic Influence on Chromatin State in Human Genome
Source: PLoS Genet. 2007 May 18;3(5):e81. doi: 10.1371/journal.pgen.0030081 (PMC1868950; doi:10.1371/journal.pgen.0030081)
Supplement: Supplementary file 10 [file pgen.0030081.st002.pdf]

oligo name	sequence
CD19_qPCR_f	GGTAGTGAGAGCTGGGATGAAAATA
CD19_qPCR_r	TCTCCAGGAACCCTCCAGACT
CD3G_qPCR_f	GGGTTCTTGCCTTCTCTCAAAG
CD3G_qPCR_r	AGCCTGTGCAGCAGCTAGACT
GAPDH_qPCR_f	CCTCCCGCTTCGCTCTCT
GAPDH_qPCR_r	GGCGACGCAAAAGAAGATG
MYOD1_qPCR_f	AGCGTTTCCCGCGGATA
MYOD1_qPCR_r	CGGCACGCCCTTTCCA
NES_qPCR_f	TCAGAAAGCCACCACCATCA
NES_qPCR_r	GCTGGAGAGTGGAACCAAAGAA
RPLP1_qPCR_f	CTAACCAGGAGATTACGAACAATGTC
RPLP1_qPCR_r	GGTTCCATGAGTTGCTTGACTTG
rs11023840-f	GTCGTCCCTGAGCACCAC
rs11023840-r	AGGGCCCACAGCAGTGTC
rs11023840-PET	(PET)-TCCTGCCCAGCAACACCCTGCCCACCTA
rs11023840-C	CGGAGACACGGGCCAGTTCTCTGC
rs11023840-T	TGGAGACACGGGCCAGTTCTCTGCAA
rs6634990-f	CGATGACTGGAACCCGAAGA
rs6634990-r	CGTGGGAATTTTCTCCCAGA
rs6634990-6FAM	(6FAM)-TCAGGGAGCCCTCTGAATAGGAGACTGAGT
rs6634990-G	GGGGAGGGAAAGGGGCTTCGCAA
rs6634990-T	TGGGAGGGAAAGGGGCTTCGCTAAT
rs2076628-f	AAGGGAGCTAGGAAACCAGGA
rs2076628-r	GAACCACGCCCAGAGCAG
rs2076628-PET	(PET)-CAGCCTCGAATTCCACGGGGTTGG
rs2076628-A	AGTTGCGCCTTTTCCAAGGCAGCCAATAC
rs2076628-G	GGTTGCGCCTTTTCCAAGGCAGCCAAT
rs220030-f	TTTTGGAAGGATTTCAAGTCTGG
rs220030-r	ACACACCACTGGCCAAACAAT
rs220030-6FAM	(6FAM)-GGTTATGGCGCATTTAACTTTTCCAAACCAGCTTTTT
rs220030-C	CGTACCGCACATAGGAAGACCTGAGGGA
rs220030-T	TGTACCGCACATAGGAAGACCTGAGGGTAA
Xba-linker	(phospho)-GCTCTAGAGC
