# Supplementary material for: Allele-Specific Chromatin Immunoprecipitation Studies Show Genetic Influence on Chromatin State in Human Genome
Source: PLoS Genet. 2007 May 18;3(5):e81. doi: 10.1371/journal.pgen.0030081 (PMC1868950; doi:10.1371/journal.pgen.0030081)
Supplement: Supplementary file 11 [file pgen.0030081.st003.pdf]

SAMPLE	FAMILY	RELATION	ID
GM10858	1347	father	1
GM10859	1347	mother	2
GM11870	1347	daughter	3
GM11871	1347	son	4
GM11872	1347	son	5
GM11875	1347	daughter	6
GM10860	1362	father	7
GM10861	1362	mother	8
GM11982	1362	daughter	9
GM11983	1362	daughter	10
GM11984	1362	son	11
GM11987	1362	son	12
GM07057	1331	father	13f
GM06990	1331	mother	14m
GM07023	1331	daughter	15d
GM07033	1331	son	16s
GM07059	1331	daughter	17d
GM06983	1331	son	18s
GM10832	1413	father	19f
GM10833	1413	mother	20m
GM12101	1413	son	21s
GM12102	1413	son	22s
GM12104	1413	daughter	23d
GM12105	1413	daughter	24d
