# Supplementary material for: Allele-Specific Chromatin Immunoprecipitation Studies Show Genetic Influence on Chromatin State in Human Genome
Source: PLoS Genet. 2007 May 18;3(5):e81. doi: 10.1371/journal.pgen.0030081 (PMC1868950; doi:10.1371/journal.pgen.0030081)
Supplement: Supplementary file 12 [file pgen.0030081.st004.pdf]

PC	A+B	A.div.AplusB
PC1	0.2576605	0.05068196
PC2	0.1341045	0.04308285
PC3	0.08469787	0.03291186
PC4	0.05057464	0.03103058
PC5	0.04033931	0.02682213
PC6	0.02709606	0.02622069
PC7	0.02436079	0.0256178
PC8	0.0181442	0.02486414
PC9	0.01551926	0.02250994
PC10	0.0139083	0.02203066
