# Supplementary material for: Allele-Specific Chromatin Immunoprecipitation Studies Show Genetic Influence on Chromatin State in Human Genome
Source: PLoS Genet. 2007 May 18;3(5):e81. doi: 10.1371/journal.pgen.0030081 (PMC1868950; doi:10.1371/journal.pgen.0030081)
Supplement: Supplementary file 13 [file pgen.0030081.st005.pdf]

PC	1331.1413	1347.1331	1347.1413	1331.1413	1347.1331	1347.1413
		A + B			A/(A+B)	
PC1	0.4037191	0.4231794	0.4160735	0.0918856	0.09665231	0.09412496
PC2	0.1171681	0.1383231	0.09018108	0.07238273	0.0792524	0.07490638
PC3	0.08861888	0.05731085	0.07133324	0.05134037	0.0539881	0.05195803
PC4	0.03002966	0.03994457	0.04029174	0.04733662	0.04580255	0.05055429
PC5	0.02447393	0.02459232	0.02715893	0.04494648	0.04399038	0.04675387
PC6	0.02232985	0.0197844	0.02436097	0.04371229	0.04275813	0.04438277
PC7	0.01946996	0.01769152	0.02036871	0.04215523	0.04051923	0.04410374
PC8	0.01899975	0.0162547	0.02010512	0.04023287	0.03941389	0.04157589
PC9	0.01783051	0.01519119	0.01956103	0.03923662	0.03798861	0.03882701
PC10	0.01546301	0.01364067	0.01940606	0.03753124	0.03664382	0.03782555
