# Supplementary material for: Allele-Specific Chromatin Immunoprecipitation Studies Show Genetic Influence on Chromatin State in Human Genome
Source: PLoS Genet. 2007 May 18;3(5):e81. doi: 10.1371/journal.pgen.0030081 (PMC1868950; doi:10.1371/journal.pgen.0030081)
Supplement: Supplementary file 14 [file pgen.0030081.st006.pdf]

			sample treatment (gDNA, input or ChIP)							
Family ID	Lymphoblastoid Cell Lines	Relation	gDNA	Input	Pol	H3Ac	H3K4	H3K9	H3K27di	H3K27tri
1347	GM10858	Father	√	√	√	√	√	√	√	√
1347	GM10859	Mother	√	√	√	√	√	√	√	√
1347	GM11870	Daugter	√	√	√	√	√	√	√	√
1347	GM11871	Son	√	√	√	√	√	√	√	√
1347	GM11872	Son	√	√	√	√	√	√	√	√
1347	GM11875	Daugter	√	√	√	√	√	√	√	√
1362	GM10860	Father	√	√	√	√	√	√	√	√
1362	GM10861	Mother	√	√	√	√	√	√	√	√
1362	GM11982	Daugter	√	√	√	√	√	√	√	√
1362	GM11983	Daugter	√	√	√	√	√	√	√	√
1362	GM11984	Son	√	√	√	√	√	√	√	√
1362	GM11987	Son	√	√	√	√	√	√	√	√
1331	GM07057	Father	√	√		√	√			
1331	GM06990	Mother	√	√		√	√			
1331	GM06983	Son	√	√		√	√			
1331	GM07023	Daugter	√	√		√	√			
1331	GM07033	Son	√	√		√	√			
1331	GM07059	Son	√	√		√	√			
1413	GM10832	Father	√	√		√	√			
1413	GM10833	Mother	√	√		√	√			
1413	GM12101	Son	√	√		√	√			
1413	GM12102	Son	√	√		√	√			
1413	GM12104	Daugter	√	√		√	√			
1413	GM12105	Daugter	√	√		√	√			
