# Supplementary material for: Allele-Specific Chromatin Immunoprecipitation Studies Show Genetic Influence on Chromatin State in Human Genome
Source: PLoS Genet. 2007 May 18;3(5):e81. doi: 10.1371/journal.pgen.0030081 (PMC1868950; doi:10.1371/journal.pgen.0030081)
Supplement: Supplementary file 15 [file pgen.0030081.st007.pdf]

Family ID	Cell Lines	Relation	Age	Gender	Race
1347	GM10858	Father	42	Male	Caucasian
1347	GM10859	Mother	41	Female	Caucasian
1347	GM11870	Daugter	21	Female	Caucasian
1347	GM11871	Son	19	Male	Caucasian
1347	GM11872	Son	18	Male	Caucasian
1347	GM11875	Daugter	13	Female	Caucasian
1362	GM10860	Father	50	Male	Caucasian
1362	GM10861	Mother	49	Female	Caucasian
1362	GM11982	Daugter	28	Female	Caucasian
1362	GM11983	Daugter	29	Female	Caucasian
1362	GM11984	Son	26	Male	Caucasian
1362	GM11987	Son	19	Male	Caucasian
1331	GM07057	Father	48	Male	Caucasian
1331	GM06990	Mother	41	Female	Caucasian
1331	GM06983	Son	11	Male	Caucasian
1331	GM07023	Daugter	21	Female	Caucasian
1331	GM07033	Son	20	Male	Caucasian
1331	GM07059	Son	18	Male	Caucasian
1413	GM10832	Father	49	Male	Caucasian
1413	GM10833	Mother	48	Female	Caucasian
1413	GM12101	Son	28	Male	Caucasian
1413	GM12102	Son	27	Male	Caucasian
1413	GM12104	Daugter	23	Female	Caucasian
1413	GM12105	Daugter	25	Female	Caucasian
