# Supplementary material for: Allele-Specific Chromatin Immunoprecipitation Studies Show Genetic Influence on Chromatin State in Human Genome
Source: PLoS Genet. 2007 May 18;3(5):e81. doi: 10.1371/journal.pgen.0030081 (PMC1868950; doi:10.1371/journal.pgen.0030081)
Supplement: Supplementary file 16 [file pgen.0030081.st008.doc]

MIAME Checklist for the manuscript entitled “Allele-specific chromatin immunoprecipitation studies show genetic influence on chromatin state in the human genome” by Kadota et al.

Experiment Design:
1.	The goal of experiment
Is to determine if genetic inheritance affects chromatin states.
2.	A brief description of the experiment
To determine if genetics affects the variations in chromatin states, we develop an allele-specific chromatin immuno-precipitation (haploChIP-on-chip) assay using the Affymetrix 10K SNP chip.  We performed a haploChIP-on-chip assays for 12 lymphoblastoid cells derived from two CEPH families using antibodies against RNA polymerase II and five post-translation modification forms of the histone H3 protein.  The use of multiple cell lines and various antibodies allowed us to evaluate variation of allelic chromatin states across these pedigrees.  Intriguingly, these studies demonstrated that chromatin state clustered by family, suggesting that inherited genetic components can determine the epigenetic state of the chromatin.
3.	Keywords
SNP, chromatin, chip, inheritance, genetic variation
4.	Experimental factors
genetic variation and antibodies
5.	Experimental design
Twenty-four CEPH lymphoblastoid cell lines from four families were used to assess the variance among individuals at active chromatin (Pol: RNA polymerase II, H3Ac: Histone H3 lysine 9/14 acetylation, H3K4: Histone H3 lysine 4 di-methylation) or inactive chromatin (H3K9: Histone H3 lysine 9 di-methylation, H3K27di: Histone H3 lysine 27 di-methylation, H3K27tri: Histone H3 lysine 27 tri-methylation).
			sample treatment (gDNA, input or ChIP)	
Family ID	Lymphoblastoid Cell Lines	Relation	gDNA	Input	Pol	H3Ac	H3K4	H3K9	H3K27di	H3K27tri	
1347	GM10858	Father	√	√	√	√	√	√	√	√	
1347	GM10859	Mother	√	√	√	√	√	√	√	√	
1347	GM11870	Daughter	√	√	√	√	√	√	√	√	
1347	GM11871	Son	√	√	√	√	√	√	√	√	
1347	GM11872	Son	√	√	√	√	√	√	√	√	
1347	GM11875	Daughter	√	√	√	√	√	√	√	√	
1362	GM10860	Father	√	√	√	√	√	√	√	√	
1362	GM10861	Mother	√	√	√	√	√	√	√	√	
1362	GM11982	Daughter	√	√	√	√	√	√	√	√	
1362	GM11983	Daughter	√	√	√	√	√	√	√	√	
1362	GM11984	Son	√	√	√	√	√	√	√	√	
1362	GM11987	Son	√	√	√	√	√	√	√	√	
1331	GM07057	Father	√	√	 	√	√	 	 	 	
1331	GM06990	Mother	√	√	 	√	√	 	 	 	
1331	GM06983	Son	√	√	 	√	√	 	 	 	
1331	GM07023	Daughter	√	√	 	√	√	 	 	 	
1331	GM07033	Son	√	√	 	√	√	 	 	 	
1331	GM07059	Son	√	√	 	√	√	 	 	 	
1413	GM10832	Father	√	√	 	√	√	 	 	 	
1413	GM10833	Mother	√	√	 	√	√	 	 	 	
1413	GM12101	Son	√	√	 	√	√	 	 	 	
1413	GM12102	Son	√	√	 	√	√	 	 	 	
1413	GM12104	Daughter	√	√	 	√	√	 	 	 	
1413	GM12105	Daughter	√	√	 	√	√	 	 	 	

6.	Quality control steps taken
Genotype call for sonicated 'input' DNA was compared to the genotype call from the genomic DNA.  The average concordance of the two genotype calls were >99% of the SNPs.  Clustering of samples by antibody type in PCA projection when PCA was analyzed with total binding intensity.
7.	Links to the publication, any supplemental websites or database accession numbers

Samples used, extract preparation and labeling:
1.	The origin of each biological sample
Lymphoblastoid cells of 24 individuals from CEPH/Utah pedigrees (family id 1347, 1362, 1331, and 1413) purchases from Coriell Cell Repositories were used in this study. 
Family ID	Cell Lines	Relation	Age	Gender	Race	
1347	GM10858	Father	42	Male	Caucasian	
1347	GM10859	Mother	41	Female	Caucasian	
1347	GM11870	Daughter	21	Female	Caucasian	
1347	GM11871	Son	19	Male	Caucasian	
1347	GM11872	Son	18	Male	Caucasian	
1347	GM11875	Daughter	13	Female	Caucasian	
1362	GM10860	Father	50	Male	Caucasian	
1362	GM10861	Mother	49	Female	Caucasian	
1362	GM11982	Daughter	28	Female	Caucasian	
1362	GM11983	Daughter	29	Female	Caucasian	
1362	GM11984	Son	26	Male	Caucasian	
1362	GM11987	Son	19	Male	Caucasian	
1331	GM07057	Father	48	Male	Caucasian	
1331	GM06990	Mother	41	Female	Caucasian	
1331	GM06983	Son	11	Male	Caucasian	
1331	GM07023	Daughter	21	Female	Caucasian	
1331	GM07033	Son	20	Male	Caucasian	
1331	GM07059	Son	18	Male	Caucasian	
1413	GM10832	Father	49	Male	Caucasian	
1413	GM10833	Mother	48	Female	Caucasian	
1413	GM12101	Son	28	Male	Caucasian	
1413	GM12102	Son	27	Male	Caucasian	
1413	GM12104	Daughter	23	Female	Caucasian	
1413	GM12105	Daughter	25	Female	Caucasian	

2.	Manipulation of biological samples and protocol used
All cells were grown in RPMI medium supplemented with 2mM L-glutamine and 15% FBS. 
3.	Experimental factor value
Twelve cell lines from family 1347 and 1362 are in a set of either experiments consisting of gDNA, Input, H3Ac, H3K4, H3K9, H3K27-di and H3K27-tri. The rest twelve from family 1331 and 1413 are in a set of three experiments consisting of gDNA, Input and H3Ac.
4.	Technical protocols for preparing the hybridization extract
The cells were chemically fixed by adding formaldehyde solution into the culture medium to a final concentration of 1%.  After centrifugation the cell pellets were rinsed twice with an ice-cold PBS solution and then suspended in a lysis buffer (all buffers used in the ChIP experiment are described in http://www.upstate.com/img/coa/17-295-33519A.pdf).  Sonication was performed on ice using Cole Parmer economical ultrasonic processor at power 9 for 12 cycles of sonication, each cycle for 10 sec followed by a 30 sec break on ice.  The cell pellets were centrifuged at 18,000 RCF (xg) for 10 min and the resulting lysates in the supernatant were stored at -80C until use.  For an immunoprecipitation experiment, the chromatin lysate was diluted by 10-fold in a ChIP dilution buffer, pre-cleared by Salmon sperm protein A agarose, and incubated with a series of antibodies overnight at 4^0^C.   The chromatin complexes were sequentially washed in low salt, high salt, LiCl salt, and TE buffers.  The protein/DNA complex was eluted in an SDS elution buffer (1% SDS, 0.1M NaHCO3).  The crosslink between protein and DNA was reversed.  The protein/DNA complex was treated with Proteinase K.  DNAs were purified using Qiagen mini-elute reaction clean-up kit.
5.	External controls
The qualities of antibodies or the qualities of ChIP-DNAs were assessed by analyzing the Paternal/Maternal allelic enrichment of ChIP-DNAs at the KvDMR (Differentially Methylated Region at imprinted LIT1 gene promoter) locus. Expected allelic enrichment (paternal allele as active vs. maternal allele as inactive) was observed at all the informative heterozygous sample for all six antibody ChIP experiment.

Hybridization procedures and parameters:
1.	The protocol and conditions used for hybridization, blocking and washing
Hybridization mix was made according to the Affymetrix GeneChip 10K protocol. Hybridization was carried out at 48C for 16hrs at 60rpm. Fluidic Station 450 was used for automate washing and staining (http://www.affymetrix.com).

Measurement data and specifications:
1.	Data
Affymetrix GeneChip Operating Software (GCOS) and GeneChip DNA Analyzing Software (GDAS) were used for chip scanning and data extraction. 
2.	Data extraction and processing protocols
All statistical analyses were developed using R and Splus packages.  The missing values in PM or MM probes were replaced by the mean MM across all SNPs.  For each SNP, we computed the ratio PM/MM and then applied the Robust Multi-array Average (RMA) method.  Probe intensity was computed by the function of max(mean(log2(PM/MM)), 0) for allele A and allele B.  The intensity at the probe set level was the average of the 10 pairs of the probes from each allele of the SNP.  

Array design:
1.	General array design
Twenty-five nucleotide oligomer probes corresponding to a perfect match of the A allele sequence (PMA) and to the B allele sequence (PMB), with another set of probes which contain a mismatch at the center of the 25-mer probes for the A allele (MMA) and for the B allele (MMB), and at sense and anti-sense orientation, total of 40 probes per SNP is used to determine the specificity of the hybridization.
2.	Array feature and reporter annotation
3.	Principal array organism
Epstein-Barr virus transformed human lymphoblastoid cells (B-Lymphocyte) from peripheral blood was used in this study.
